# Synthesis of (−)-julocrotine and a diversity oriented Ugi-approach to analogues and probes

**DOI:** 10.3762/bjoc.7.175

**Published:** 2011-11-07

**Authors:** Ricardo A W Neves Filho, Bernhard Westermann, Ludger A Wessjohann

**Affiliations:** 1 Department of Bioorganic Chemistry, Leibniz Institute of Plant Biochemistry, Weinberg 3, 06120 Halle, Germany; 2 Martin-Luther-University Halle-Wittenberg, Institute of Organic Chemistry, Kurt-Mothes-Str. 2, 06120 Halle, Germany

**Keywords:** diversity oriented synthesis, julocrotine, leishmania, Mitsunobu reaction, Ugi reaction

## Abstract

An improved total synthesis of (−)-julocrotine in three steps from Cbz-glutamine, in 51% overall yield, is presented. To demonstrate the potential of the heterocyclic moiety for diversity oriented synthesis, a series of (−)-julocrotine analogues was synthesized by employing the heterocyclic precursor as an amino input in Ugi four-component reactions (Ugi-4CR) [1].

## Introduction

Julocrotine (**1**) is a natural glutarimide alkaloid isolated from several plants of the genus Croton [2–4], including *Croton cuneatus* Klotzsch, which is used by Amazonia natives in anti-inflammatory and analgesic medicines. The structure of this glutarimide-containing alkaloid was first proposed in 1960, based upon a series of degradative experiments, but only confirmed in 2008 by X-ray analysis [5–7]. Most interestingly, it was found to inhibit the growth of promastigote and amastigote forms of the protozoan *Leishmania amazonensis (L.)* with no cytotoxicity against the host cell [8]. This parasite causes cutaneous leishmaniasis, a neglected disease that affects more than 12 million people in tropical countries [9].

In addition, the glutarimide motif can be considered as a privileged structure. Compounds with this pharmacophore often exhibit a wide range of biological properties including anti-inflammatory [10], antitumor [11–12], and anticonvulsive properties [13].

**Figure 1:**
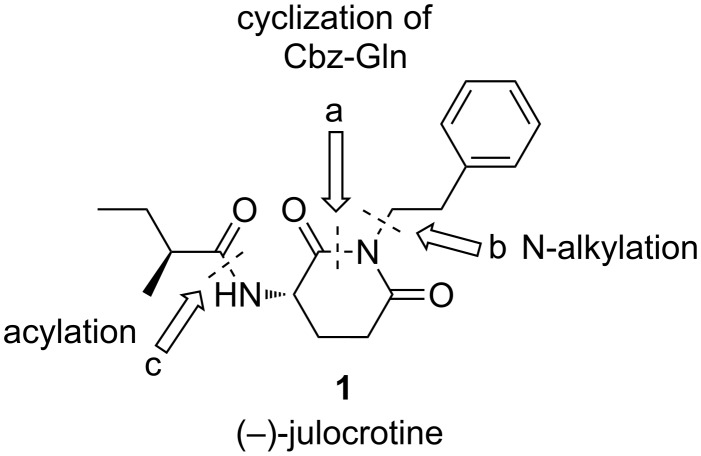
Retrosynthetic scheme for (−)-julocrotine (**1**).

Because of the low yields of julocrotine obtained through isolation from natural sources and the necessity to gain access to larger quantities of this substance for further biological screening, Silva and Joussef developed a straightforward total synthesis in six steps [14]. Starting from L-glutamic acid, their chiral-pool approach yielded the desired optically active natural product in 41% overall yield. After analyzing the structure of (−)-julocrotine, we set out to synthesize it in only three steps from commercially available L-Cbz-glutamine, in a sequence of cyclization (a), N-alkylation (b), and the removal of the protecting group followed by acylation with (*S*)-2-methylbutanoic acid (c) [15] (Figure 1).

Based on this flexible route, we also envisioned the synthesis of derivatives utilizing post-cyclization transformations by multicomponent reactions. This diversity-driven approach benefits from the fact that the heterocyclic moiety may be considered a privileged structural element for bioactivity.

## Results and Discussion

**Scheme 1:**
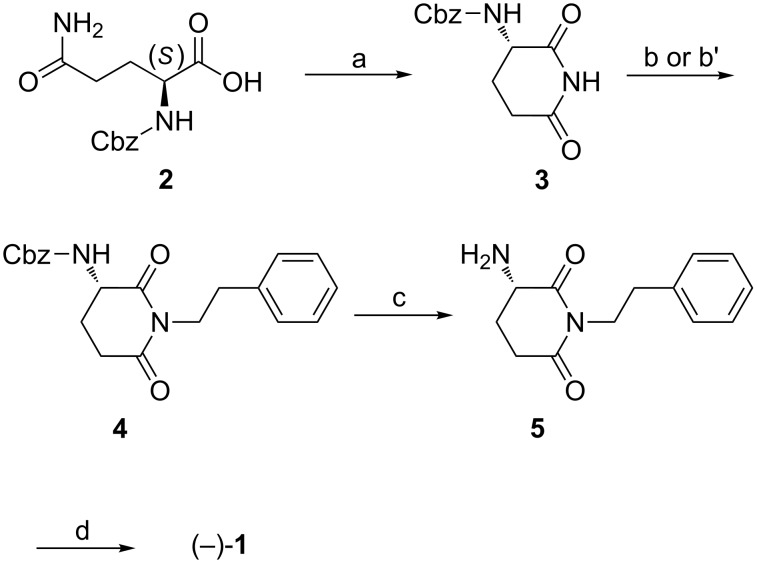
Reactions and conditions: (a) DCC, NHS, DMF 80 °C, 18 h, 76%. (b) Ph(CH_2_)_2_Br, K_2_CO_3_, acetone, r.t., 20 h, (±)**-4**, 98%. (b') Ph(CH_2_)_2_OH, DIAD, PPh_3_, THF, r.t., 20 h, (−)-**4**, 90%. (c) H_2_, 10% w/w Pd/C, MeOH, r.t., 4 h, quant. (d) (*S*)-2-methylbutanoic acid, EDCl, HOBt, CH_2_Cl_2_, r.t., 16 h, 73%.

The synthetic approach, illustrated in Scheme 1, starts from Cbz-glutamine **2**, which reacted in the presence of dicyclohexylcarbodiimide (DCC) and *N*-hydroxysuccinimide (NHS) in DMF to afford Cbz-glutarimide **3** in 76% yield in optically pure form [16]. To alkylate the imide-moiety, glutarimide **3** was reacted with phenylethyl bromide in the presence of potassium carbonate at room temperature. The desired compound **4** was obtained in 98% isolated yield, but analysis revealed racemization. Indeed, the equilibration at the chiral center of **4** can be observed even in the presence of weak bases such as potassium carbonate [17]. Thus, we decided to use a base-free N-alkylation protocol, namely the Mitsunobu reaction of **3** and the readily available 2-phenylethanol [18]. This protocol gave the desired optically active product in 90% yield ([α]^20^_D_ −29.2). The key intermediate **4** was hydrogenated on Pd/C at room temperature to afford **5**, which was coupled with (*S*)-2-methylbutanoic acid in the presence of EDCl and HOBt to afford (−)-julocrotine (**1**) in 73% yield, over two steps. The HRMS, ^1^H and ^13^C NMR spectra, optical rotation, and melting point of **1** were consistent with the reported data [2,14–15].

**Scheme 2:**
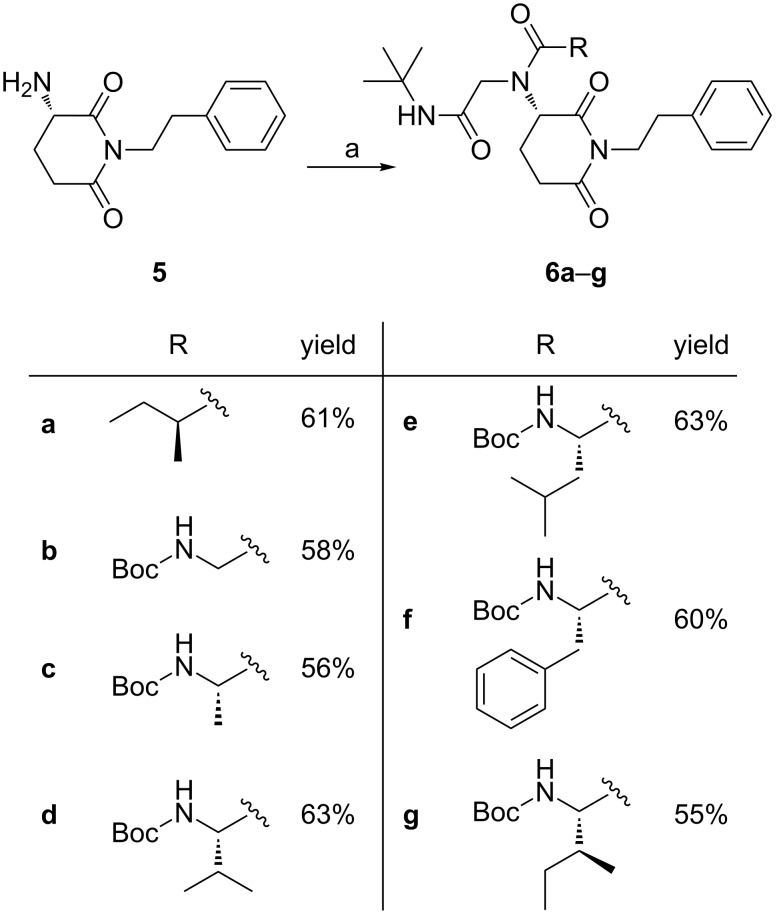
Reactions and conditions: (a) (CH_2_O)*_n_*, MeOH, r.t., 2 h then, RCOOH and *t*-BuNC, r.t., 18 h.

For the diversity oriented synthesis the advanced intermediate **5** was used as the amino component in an Ugi-4CR with (*S*)-2-methylbutanoic acid, hydrophobic amino acids, formaldehyde and *tert*-butyl isocyanide (Scheme 2). These analogues possess a protease-resistant peptoid scaffold and this might lead to an enhanced activity [19–20]. In this endeavor, all Ugi reactions were initiated by pre-imine formation of **5** and reaction with formaldehyde as the oxo-component, after which the multicomponent reaction was completed by the addition of (*S*)-2-methylbutanoic acid, Boc-Gly, Boc-Ala, Boc-Val, Boc-Leu, Boc-Phe and Boc-Ile and *tert*-butyl isocyanide. Following this procedure, the desired optically active compounds **6a–g** were obtained in 55–63% yields. Their structures were confirmed by ^1^H, ^13^C NMR and HRMS spectra.

**Scheme 3:**
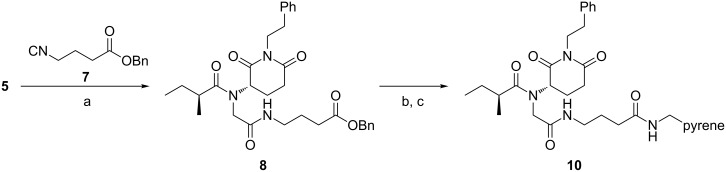
Reactions and conditions: (a) (CH_2_O)*_n_*, MeOH, r.t., 2 h then, (*S*)-2-methylbutanoic acid and **7**, r.t. 18 h, 61%. (b) H_2_, 10% w/w Pd/C, MeOH, r.t., 10 h. (c) 1-pyrenemethylamine hydrochloride, Et_3_N, EDCl, DMAP, CH_2_Cl_2_, r.t., 24 h, 80% over two steps.

Finally, the Ugi-4CR was utilized for the synthesis of a molecular probe prototype of **1**, which can be used for intercalation studies (Scheme 3). For this propose, the natural product scaffold should be attached through a spacer to a reporter tag, which is normally a luminescent group or a dye. The advanced intermediate **5** was converted to the respective imine as depicted in Scheme 2 and then reacted with (*S*)-2-methylbutanoic acid and isonitrile **7** to afford the intermediate **8** in 61% yield. This compound was then hydrogenated to afford **9** and then directly coupled with 1-pyrenemethylamine, by using EDCl as coupling reagent, to yield the designed probe prototype **10** in 80% yield (from **8**).

Pyrene derivative **10** exhibited strong blue luminescence in both solution and solid phase. This probe may be used for tracking the (−)-julocrotine in biological systems, in particular in promastigote and amastigote forms of protozoan *Leishmania amazonensis (L.)*. It could be helpful to elucidate the to-date unknown mode of action of this natural product in the parasite.

## Conclusion

In summary, a highly efficient method to synthesize (−)-julocrotine (**1**) in three steps from Cbz-glutamine **2** was developed. The approach affords the natural product in 51% overall yield. The versatility of the developed protocol was demonstrated in the synthesis of seven julocrotine analogues and a molecular probe utilizing Ugi-4CRs. The desired compounds **6a–g** and **10** were obtained in good yields.

## Supporting Information

File 1 Experimental procedures and analytical data.
