# Supplementary material for: Synthesis of (−)-julocrotine and a diversity oriented Ugi-approach to analogues and probes
Source: Beilstein J Org Chem. 2011 Nov 7;7:1504–7. doi: 10.3762/bjoc.7.175 (PMC3252850; doi:10.3762/bjoc.7.175)
Supplement: File 1 — Experimental procedures and analytical data. [file Beilstein_J_Org_Chem-07-1504-s001.pdf]

Supporting Information

for

Synthesis of (−)-julocrotine and a diversity oriented Ugi-approach to analogues and probes

Ricardo A. W. Neves Filho[1], Bernhard Westermann[1,2] and Ludger A. Wessjohann[*§,1,2]

Address: [1]Department of Bioorganic Chemistry, Leibniz Institute of Plant Biochemistry, Weinberg 3, 06120 Halle, Germany and [2]Martin-Luther-University Halle-Wittenberg, Institute of Organic Chemistry, Kurt-Mothes-Str. 2, 06120 Halle, Germany

Email: Ludger A. Wessjohann - wessjohann@ipb-halle.de

[*]Corresponding author

[§]Tel: +49 345 5582 1301; Fax: +49 345 5582 1309

Experimental procedures and analytical data

General remarks

All commercially available chemicals were used without further purification. Dichloromethane and THF were dried before use, following conventional procedures. HPLC grade methanol was used in Ugi reactions. Analytical thin layer chromatography (TLC) was performed using silica gel 60 F_{254} aluminum sheets and the visualization of the spots was done under UV light (254 nm) or by reaction with a solution of ninhydrin in *n*-butanol (3:1 w/v), 3% acetic acid and heating. Flash column chromatography was performed over silica gel (0.040–0.063 mm). Melting points are uncorrected. ^{1}H and ^{13}C NMR were recorded in $CDCl_3$ solutions at 25 °C, at 400 MHz and 100 MHz,

respectively. Chemical shifts (δ) are reported in ppm relative to the TMS (^{1}H NMR) and to the solvent signal (^{13}C NMR). High resolution ESI mass spectra were obtained from a Bruker Apex III Fourier transform ion cyclotron resonance (FT-ICR) mass spectrometer equipped with an Infinity cell, a 7.0 Tesla superconducting magnet, an RF-only hexapole ion guide and an external electrospray ion source (off-axis spray).

(*S*)-Benzyl (2,6-dioxopiperidin-3-yl)carbamate (**3**) [1].

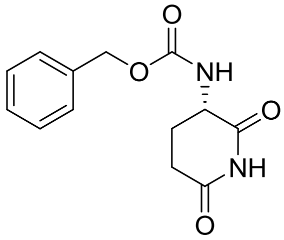

To a stirred solution of Cbz-L-Glutamine (12.0 g, 42.9 mmol) in dry DMF (120 mL) was added DCC (9.75 g, 47.3 mmol) and *N*-hydroxysuccinimide (5.44 g, 47.3), and the mixture was heated at 80 °C for 18 h. The reaction mixture was cooled to r.t. and the precipitated DCU was filtered off. The filtrate was diluted with EtOAc (50 mL), washed with water (3 x 50 mL) and brine (50 mL), and dried over anhydrous Na_2SO_4. The solvent was evaporated under reduced pressure and the crude material purified by silica gel isocratic column chromatography, with (3:7) ethyl acetate:dichloromethane as eluents, to afford 8.53 g of **3** as a white solid.

Yield: 76%, mp 111–112 °C (lit. [2]: mp 113.2 °C; $[\alpha]_D^{20}$ −46.6 (*c* 1.0, MeOH), (lit. [2]: $[\alpha]_D^{20}$ −42.7 (*c* 1.0, MeOH)); ^{1}H NMR δ 1.89 (dq, *J* = 5.2, 13.6 Hz, 1H), 2.58 (m, 1H), 2.69 (m, 1H), 2.79 (m, 1H), 4.35 (q, *J* = 5.2 Hz, 1H), 5.11 (s, 2H), 5.82 (d, *J* = 6.4 Hz, 1H), 7.29–7.38 (m, 5H), 8.93 (s, 1H); ^{13}C NMR δ 25.0, 31.1, 51.9, 67.2, 128.09, 128.5, 135.9, 156.1, 171.7, 172.1; ESI-MS *m/z* 263.2 [M +1], 285.3 [M + 23].

(S)-Benzyl (2,6-dioxo-1-phenethylpiperidin-3-yl)carbamate (**4**) [3].

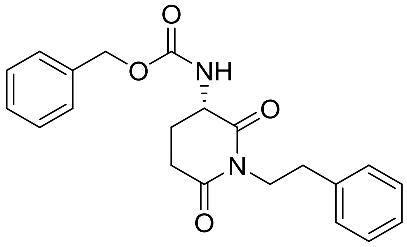

To a stirred solution of **3** (7.54 g, 28.8 mmol), 2-phenylethanol (2.67 mL, 22.2 mmol) and triphenylphosphine (7.54 g, 28.8 mmol) in anhydrous THF (240 mL) was added dropwise DIAD (6.09 mL, 31.0 mmol) at r.t. After the mixture had been stirred for 20 h, the solvent was removed under reduced pressure. The crude material was purified by silica gel isocratic column chromatography, with (3:7) ethyl acetate:hexanes as eluents, to afford 7.29 g of **4** as colorless crystals.

Yield: 90%; mp 122–123 °C (lit. [4]: mp 122–123 °C); $[\alpha]_D^{20}$ −29.2 (*c* 1.0, $CHCl_3$) (lit. [4]: $[\alpha]_D^{20}$ −30.6 (*c* 1.12, $CHCl_3$)); ^{1}H NMR δ 1.74 (dq, *J* = 4.8, 13.2 Hz, 1H), 2.48 (m, 1H), 2.67 (m, 1H), 2.81 (m, 3H), 4.02 (m, 2H), 4.27 (m, 1H), 5.15 (s, 2H), 5.64 (br, 1H), 7.20–7.33 (m, 10H); ^{13}C NMR δ 25.5, 32.1, 35.4, 41.4, 52.6, 69.3, 126.3, 128.1, 128.3, 128.5, 128.9, 136.0, 138.9, 158.5, 171.4, 172.9; ESI-MS *m*/*z* 367.3 [M +1], 389.5 [M + 23].

(S)-*N*-[(S)-2,6-Dioxo-1-phenethylpiperidin-3-yl]-2-methylbutanamide, (−)-julocrotine (**1**).

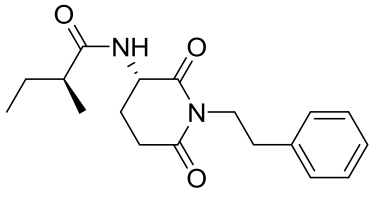

To a stirred solution of compound **4** (0.37 g, 1.0 mmol) in MeOH (10 mL) was added Pd/C (34.0 mg, 10% w/w). The reaction vessel was evacuated, purged with hydrogen and kept under H_2 atmosphere (balloon). The suspension was stirred for 4 h at r.t. After filtration through Celite, the solvent was removed under reduced pressure to yield an oily product **5**, which was used in the next step without further purification [5].

To a solution of crude intermediate **5** in CH_2Cl_2 (2 mL) were added (*S*)-2-methylbutanoic acid (0.12 mL, 1.1 mmol), EDCl (0.23 g, 1.2 mmol) and HOBt (0.15 g, 1.1 mmol). After the mixture had been stirred for 16 h, the solvent was removed under reduced pressure. The crude material was purified by silica gel gradient column chromatography, with (3:7–1:1) ethyl acetate:hexanes as eluents, to afford 0.24 g of **1** as a white solid.

Yield: 73%; mp 107–108 °C (lit. [4]: mp 106–107°C); $[\alpha]_D^{20}$ −44.02 (*c* 0.88, MeOH) (lit. [4]: $[\alpha]_D^{20}$ −46.0 (*c* 0.88, MeOH)); ^{1}H NMR δ 0.93 (t, *J* = 7.4 Hz, 3H), 1.16 (d, *J* = 6.8 Hz, 3H), 1.47 (m, 1H), 1.68 (m, 2H), 2.22 (m, 1H), 2.50 (m, 1H), 2.68, 2.76 (d, *J* = 5.2, 3.2 Hz, 2H), 2.80 (t, *J* = 7.6 Hz, 2H), 4.00 (m, 2H), 4.49 (ddd, *J* = 5.2, 5.2, 13.2 Hz, 1H), 6.35 (d, *J* = 5.2 Hz, 1H), 7.18–7.29 (m, 5H); ^{13}C NMR δ 11.7, 17.2, 24.3, 27.1, 31.6, 33.8, 41.5, 42.8, 51.1, 126.5, 128.4, 128.9, 138.0, 170.9, 171.8, 176.8.

General procedure for the synthesis of compounds 6a–g.

To a solution of compound **5** (0.23 g, 1.0 mmol) in MeOH (5.0 mL) was added paraformaldehyde (30 mg, 1.0 mmol), and the contents were stirred for 2 h [6]. After this time the suitable carboxylic acid (1.0 mmol) and *tert*-butyl isonitrile (0.11 mL, 1.0 mmol) were added, and the stirring was continued for 18 h. The solvent was removed under reduced pressure and the crude material purified by silica gel column

chromatography to afford the desired products. The details for the purification of the individual products are given below.

(*S*)-*N*-(2-(*tert*-Butylamino)-2-oxoethyl)-*N*-((*S*)-2,6-dioxo-1-phenethylpiperidin-3-yl)-2-methylbutanamide (**6a**).

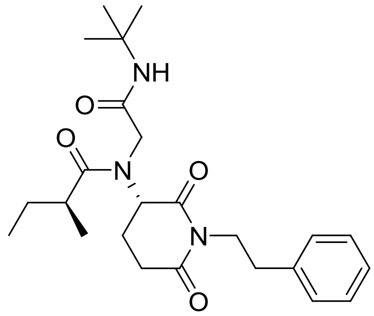

Purified by silica gel isocratic column chromatography with (3:7) ethyl acetate:hexanes as eluents. Yield: 61%; $[\alpha]_D^{20}$ −3.5 (*c* 1.0, MeOH); ^{1}H NMR δ 0.85 (t, *J* = 7.6 Hz, 3H), 1.08 (d, *J* = 6.4 Hz, 2H), 1.38 (m, 10H), 1.58 (m, 1H), 1.96 (m, *J* = 6.4 Hz, 1H), 2.44 (m, 3H), 2.83 (m, 3H), 3.65 (m, 2H), 4.01 (m, 2H), 4.21(1H, m), 7.16–7.28 (m, 5H), 8.41 (bs, 1H); ^{13}C NMR δ 11.5, 17.1, 20.7, 27.3, 28.5, 31.3, 33.5, 37.3, 41.5, 51.6, 126.3, 128.3, 128.43, 138.5, 167.4, 170.4, 171.0; HRMS *m*/*z* calcd for $C_{24}H_{35}N_3NaO_4$, 452.2525; found, 452.2519.

(*S*)-*tert*-Butyl 2-((2-(*tert*-butylamino)-2-oxoethyl) (2,6-dioxo-1-phenethylpiperidin-3-yl)amino)-2-oxoethylcarbamate (**6b**).

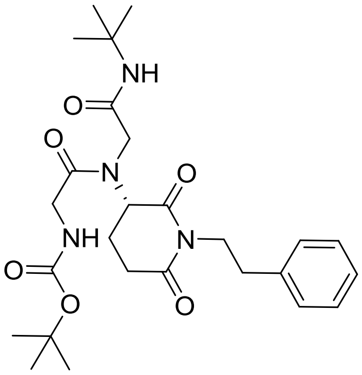

Purified by silica gel isocratic column chromatography with (1:1) ethyl acetate:hexanes as eluents. Yield: 58%; $[\alpha]_D^{20}$ −16.4 (*c* 1.0 MeOH); ^{1}H NMR δ 1.33 (m, 18H), 1.97 (m, 1H), 2.37 (m, 1H), 2.50 (m, 1H), 2.70–2.77 (m, 3H), 3.69 (m, 2H), 3.87–3.98 (m, 5H), 5.31 (bs, 1H), 7.08–7.23 (m, 5H), 8.00 (bs, 1H); ^{13}C NMR δ 20.5, 28.1, 28.3, 31.2, 33.5, 40.5, 41.4, 42.1, 51.6, 60.1, 79.6, 126.3, 128.3, 128.7, 138.2, 155.4, 166.3, 170.3, 170.7; HRMS *m*/*z* calcd for $C_{26}H_{38}N_4NaO_6$, 525.2689; found, 525.2684.

tert-Butyl (*S*)-1-((2-(*tert*-butylamino)-2-oxoethyl) ((*S*)-2,6-dioxo-1-phenethylpiperidin-3-yl)amino)-1-oxopropan-2-yl carbamate (**6c**).

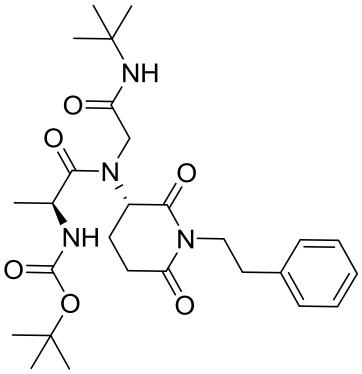

Purified by silica gel isocratic column chromatography with (1:1) ethyl acetate:hexanes as eluent. Yield: 56%; mp 149–150 °C; $[\alpha]_D^{20}$−55.6 (*c* 1.0 MeOH); ^{1}H NMR δ 1.26 (m, 3H), 1.35 (m, 18H), 1.98 (m, 1H), 2.38 (m, 1H), 2.54 (m, 1H), 2.75–2.79 (m, 3H), 3.69

(m, 1H), 3.94–4.03 (m, 4H), 4.33 (m, 4H), 5.20 (bd, 1H), 7.13–7.25 (m, 5H), 7.92 (bs, 1H); ^{13}C NMR δ 17.6, 20.4, 28.1, 28,3, 31.3, 33.5, 40.5, 41.4, 46.8, 51.8, 60.2, 79.7, 126.3, 128.3, 128.8, 138.3, 155.0, 166.6, 167.0, 170.4; HRMS *m*/*z* calcd for $C_{27}H_{40}N_4NaO_6$, 539.2846; found, 539.2827.

tert-Butyl (*S*)-1-((2-(*tert*-butylamino)-2-oxoethyl) ((*S*)-2,6-dioxo-1-phenethylpiperidin-3-yl)amino)-3-methyl-1-oxobutan-2-yl carbamate (**6d**).

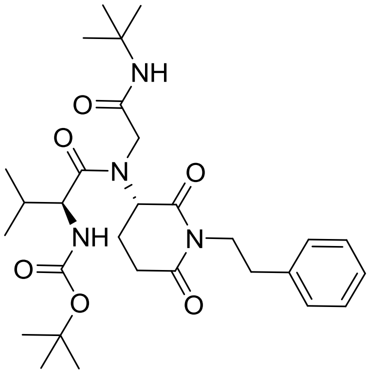

Purified by silica gel isocratic column chromatography with (3:7) ethyl acetate:hexanes as eluent. Yield: 63%; $[\alpha]_D^{20}$ −41.6 (*c* 1.0 MeOH); ^{1}H NMR δ 0.91–1.00 (m, 6H), 1.44 (m, 18H), 1.83–2.07 (m, 2H), 2.38–2.60 (m, 2H), 2.82 (m, 3H), 3.62–3.75 (m, 2H), 3.98–4.02 (m, 3H), 4.22 (m, 1H), 5.01 (d, *J* = 9.2 Hz, 1H), 7.18–7.28 (m, 5H), 8.08 (bs, 1H); ^{13}C NMR δ 17.5, 19.5, 20.5, 25.6, 28.3, 28.5, 31.2, 31.5, 33.7, 41.7, 52.6, 55.5, 60.2, 67.9, 79.9, 126.5, 128.5, 128.9, 138.6, 155.7, 170.0, 170.5; HRMS *m*/*z* calcd for $C_{29}H_{44}N_4NaO_6$, 567.3159; found, 567.3153.

tert-Butyl (*S*)-1-((2-(*tert*-butylamino)-2-oxoethyl) ((*S*)-2,6-dioxo-1-phenethylpiperidin-3-yl)amino)-4-methyl-1-oxopentan-2-ylcarbamate (**6e**).

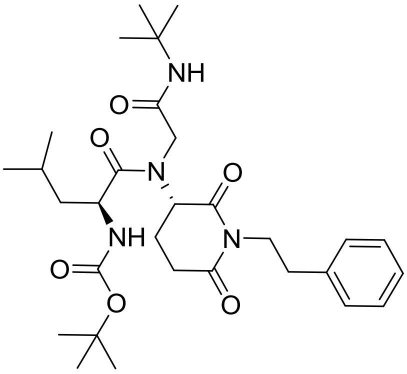

Purified by silica gel isocratic column chromatography with (3:7) ethyl acetate:hexanes as eluent. Yield: 63%; $[\alpha]_D^{20}$ −52.1 (*c* 1.0 MeOH); ^{1}H NMR δ 0.86–0.91 (m, 6H), 1.34 (m, 20H), 1.66 (m, 1H), 1.96 (m, 1H), 2.29 (m, 1H), 2.50 (m, 1H), 2.74 (m, 3H) 3.71 (m, 1H), 3.92 (t, *J* = 8.4 Hz, 2H), 4.01 (m, 2H), 4.29 (m, 1H), 4.96 (d, *J* = 8.4 Hz, 1H), 7.11–7.22 (m, 5H), 7.95 (bs, 1H); ^{13}C NMR δ 20.4, 21.4, 23.3, 24.5, 28.1, 28.3, 28.5, 31.4, 33.6, 41.2, 41.5, 49.5, 51.9, 60.2, 79.8, 126.3, 128.3, 128.8, 138.4, 155.5, 166.6, 169.8, 170.5; HRMS *m*/*z* calcd for $C_{30}H_{46}N_4NaO_6$, 581.3315; found, 581.3309.

tert-Butyl (*S*)-1-((2-(*tert*-butylamino)-2-oxoethyl) ((*S*)-2,6-dioxo-1-phenethylpiperidin-3-yl)amino)-1-oxo-3-phenylpropan-2-yl carbamate (**6f**).

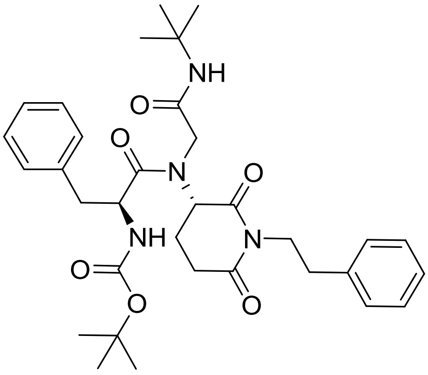

Purified by silica gel isocratic column chromatography with (3:7) ethyl acetate:hexanes as eluents. Yield: 60%; $[\alpha]_D^{20}$ −63.5 (*c* 1.0 MeOH); ^{1}H NMR δ 1.35 (m, 18H), 1.79 (m,

1H), 2.46 (m, 2H), 2.82 (m, 3H), 2.87–3.01 (m, 2H), 1.69 (m, 2H), 3.96 (m, 3H), 4.57 (m, 1H), 5.15 (br, 1H), 7.17–7.28 (m, 10H), 7.99 (bs, 1H); ^{13}C NMR δ 20.1, 28.2, 28.3, 31.4, 33.6, 39.3, 41.5, 51.8, 60.3, 80.0, 126.4, 127.3, 128.4, 128.5, 128.8, 129.4. 135.4, 138.5, 154.9, 166.4, 169.7, 170.4, 172.1; HRMS *m*/*z* calcd for $C_{33}H_{44}N_4NaO_6$, 615.3159; found, 615.3153.

tert-Butyl (2*S*,3*S*)-1-((2-(*tert*-butylamino)-2-oxoethyl) ((*S*)-2,6-dioxo-1-phenethyl piperidin-3-yl)amino)-3-methyl-1-oxopentan-2-yl carbamate (**6g**).

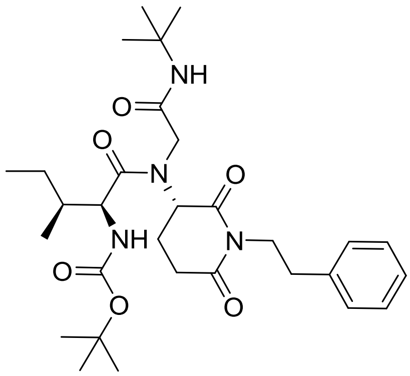

Purified by silica gel isocratic column chromatography with (3:7) ethyl acetate:hexanes as eluent. Yield: 55%; $[\alpha]_D^{20}$ −52.77 (*c* 1.0 MeOH); ^{1}H NMR δ 0.84–093 (m, 6H), 1.48 (m, 1H), 1.37 (m, 19H), 1.55–1.64 (m, 1H), 2.03 (m, 1H), 2.49 (m, 2H), 2.79 (m, 3H), 3.79 (m, 1H), 3.98 (m, 3H), 4.23 (m, 2H), 5.01 (d, *J* = 8.4 Hz, 1H), 7.15–7.27 (m, 5H), 7.98 (bs, 1H); ^{13}C NMR δ 11.2, 15.5, 20.3, 24.1, 28.2, 28.3, 28.4, 31.4, 33.6, 37.8, 41.5, 51.9, 54.7, 60.2, 79.8, 126.3, 128.3, 128.8, 138.5, 155.6, 166.6, 169.7, 170.5, 172.6; HRMS *m*/*z* calcd for $C_{30}H_{46}N_4NaO_6$, 581.3315; found, 581.3309.

Preparation of isocyanide 7 from γ-aminobutyric acid

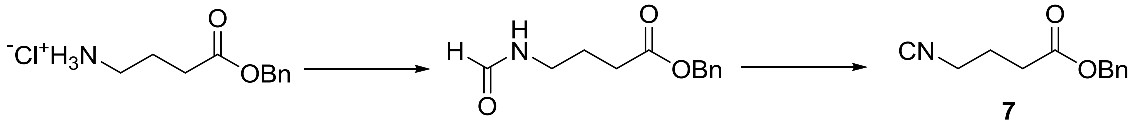

Benzyl 4-aminobutyrate, HCl salt

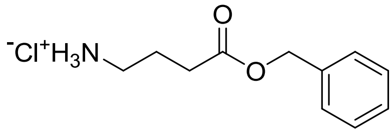

To a stirred suspension of γ-aminobutyric acid (10.3 g, 0.1 mol) in benzyl alcohol (150 mL) at 0 °C was added dropwise thionyl chloride (73 mL, 1.0 mol) over 1 h. The resulting solution was heated at 80 °C for 4 h and then allowed to cool down overnight. The contents were poured into diethyl ether (1.5 L) and then stored at −30 °C to allow for complete precipitation. The precipitated solid was filtered off, washed with diethyl ether, and recrystallized from cold diethyl ether/ethanol (9:1) to afford 13.3 g of the product as colorless needles.

Yield: 58%; mp 108–109 °C (lit. [7]: mp 109–110 °C); ^{1}H NMR δ 2.03 (q, *J* = 7.6 Hz, 2H,), 2.45 (t, *J* = 7.6 Hz, 2H), 3.03 (m, 2H), 4.25 (s, 3H), 5.01 (s, 2H), 7.24 (m, 5H); ^{13}C NMR δ 22.4, 30.9, 39.1, 66.5, 126.9, 128.2, 128.5, 135.6, 172.9.

Benzyl 4-formamidobutanoate

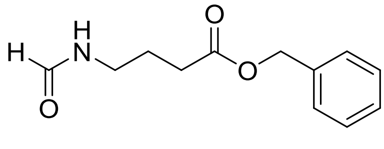

To benzyl 4-aminobutyrate HCl salt (13.3 g, 57.9 mmol) was added trimethyl orthoformate (150 mL) at r.t. and the mixture was stirred under reflux for 18 h. The contents were then allowed to cool down to r.t. and the solvent was removed under

reduced pressure. Further co-evaporations with toluene (25 mL, 2 times) were performed in order to remove remaining traces of trimethyl orthoformate. The product was obtained as a colorless oil and used in the next step without further purification.

Yield: quant.; ^{1}H NMR δ 1.87 (q, *J* = 6.8 Hz, 2H), 2.42 (t, *J* = 6.8 Hz, 2H), 3.32 (q, *J* = 6.8 Hz, 2H), 5.01 (s, 2H), 6.20 (bs, 1H), 7.34 (5H, m), 8.11 (s, 1H); ^{13}C NMR δ 24.3, 31.6, 37.6, 66.4, 126.9, 128.2, 128.4, 135.6, 161.7, 173.1; HRMS *m*/*z* calcd for $C_{12}H_{15}NO_3Na$, 244.0950; found, 244.0944.

Benzyl 4-isocyanobutanoate (**7**)

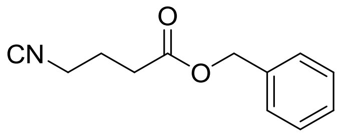

To a solution of benzyl 4-formamidobutanoate (12.2 g, 55.0 mmol) and diisopropylamine (23.3 mL, 165.0 mmol) in dichloromethane (200 mL) at 0 °C was added phosphoryl chloride (6.13 mL, 66.0 mmol) dropwise for 1 h under a nitrogen atmosphere. The solution was allowed to warm to r.t. and stirred for 4 h. Aqueous $NaHCO_3$ solution (100 mL) was added to the reaction and the contents transferred to a separatory funnel. The organic layer was successively washed with concentrated $NaHCO_3$ (two times, 100 mL), brine (once, 100 mL) and dried over anhydrous Na_2SO_4. The solvent was removed under reduced pressure and the crude product purified by isocratic column chromatography on silica gel, with (3:7) ethyl acetate:hexanes as eluent, to give 8.0 g of the title compound as light-yellow oil.

Yield: 72%; ^{1}H NMR δ 2.01 (m, 2H), 2.56 (t, *J* = 7.2 Hz, 2H), 3.32 (tt, *J* = 6.8, 1.6 Hz, 2H), 5.14 (s, 2H), 7.36 (5H, m); ^{13}C NMR δ 24.1, 30.4, 40.7, 66.6, 128.2, 128.3, 128.5, 135.5, 156.7, 171.9; HRMS *m*/*z* calcd for $C_{12}H_{13}NO_2Na$, 226.0844; found, 226.0838.

Benzyl 4-(2-((*S*)-*N*-((*S*)-2,6-dioxo-1-phenethylpiperidin-3-yl)-2-methylbutanamido) acetamido) butanoate (**8**).

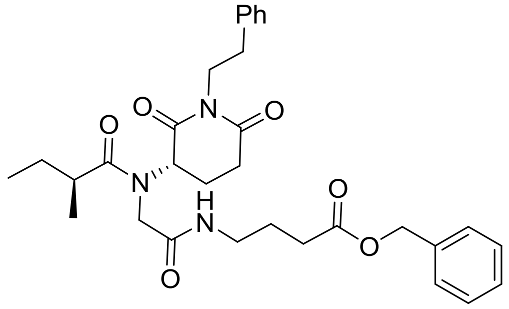

To a stirred solution of compound **5** (0.37 g, 1.0 mmol) in MeOH (10 mL), paraformaldehyde (30 mg, 1.0 mmol) was added, and the contents were stirred for 2 h. Then (*S*)-2-methylbutanoic acid (0.11 mL, 1.0 mmol) and isonitrile **7** (0.20 g, 1.0 mmol) were added and the stirring was continued for 18 h. The solvent was removed under reduced pressure and the crude material purified by gradient silica gel column chromatography with (100:0–95:5) ethyl acetate:MeOH as eluent. The obtained material was dissolved in ethyl acetate (10 mL), filtered through a syringe filter (0.45 micron pore size) and evaporated to afford 0.34 g of the target compound **8** as a light-yellow oil.

Yield:61%; $[\alpha]_D^{20}$ −3.02 (*c* 1.0, $CHCl_3$); ^{1}H NMR δ 0.87 (t, *J* = 7.4 Hz, 3H), 1.07 (d, *J* = 6.8 Hz, 3H), 1.41 (m, 1H), 1.59 (m, 1H), 1.95 (m, 3H), 2.45 (m, 6H), 2.83 (t, *J* = 7.6 Hz, 2H), 3.36 (q, *J* = 6.8 Hz, 2H), 3.73 (m, 2H), 4.01 (t, *J* = 7.6 Hz, 2H), 4.6 (s, 1H), 5.09 (s, 2H), 7.17–7.35 (m, 10H), 8.83 (bs, 1H); ^{13}C NMR δ 11.4, 16.8, 20.5, 24.4, 27.2, 31.2, 31.4, 33.5, 36.9, 38.7, 41.6, 52.6, 60.2, 66.1, 126.2, 128.0, 128.1, 128.4, 128.6, 128.8, 135.6, 138.3, 168.4, 170.3, 171.6, 172.5; HRMS *m*/*z* calcd for $C_{31}H_{39}N_3NaO_6$, 572.2737; found, 572.2731.

4-(2-((*S*)-*N*-((*S*)-2,6-Dioxo-1-phenethylpiperidin-3-yl)-2-methylbutanamido) acetamido) butanoic acid (**9**).

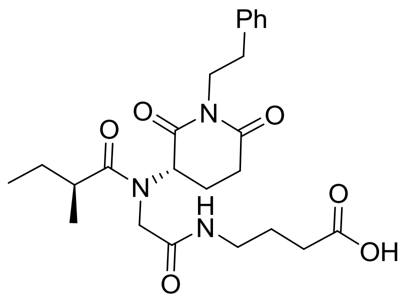

To a stirred solution of **8** (0.27 g, 0.5 mmol) in MeOH (5 mL) was added Pd/C (26 mg, 10% w/w). The reaction vessel was evacuated, purged with hydrogen and kept under H_2 atmosphere (balloon). The suspension was stirred for 4 h at r.t. and filtered through Celite to remove the heterogeneous catalyst. The solvent was evaporated under reduced pressure to yield 0.23 g of a colorless oil, which was used in the next step without further purification.

Yield: quant.; $[\alpha]_D^{20}$ −7.02 (*c* 1.0, $CHCl_3$); ^{1}H NMR δ 0.86 (t, *J* = 7.4 Hz, 3H), 1.08 (d, *J* = 6.8 Hz, 3H), 1.42 (m, 1H), 1.59 (m, 1H), 1.85 (m, 2H),1.93 (m, 1H) , 2.47 (m, 6H), 2.85 (t, *J* = 7.6 Hz, 2H), 3.37 (q, *J* = 6.8 Hz, 2H), 3.73 (m, 2H), 4.05 (t, *J* = 7.6 Hz, 2H), 4.6 (s, 1H), 7.25 (m, 5H), 8.93 (bs, 1H); ^{13}C NMR δ 11.5, 16.9, 20.6, 24.2, 27.3, 31.2, 31.3, 33.5, 37.1, 38.9, 41.7, 52.8, 60.2, 126.3, 128.4, 128.7, 138.5, 169.3, 170.5, 171.9, 176.5; HRMS *m*/*z* calcd for $C_{24}H_{33}N_3NaO_6$, 482.2267; found, 482.2261.

(*S*)-*N*-((*S*)-2,6-Dioxo-1-phenethylpiperidin-3-yl)-2-methyl-*N*-(2-oxo-2-(4-oxo-4-(pyren-1-ylmethylamino) butylamino) ethyl) butanamide (**10**).

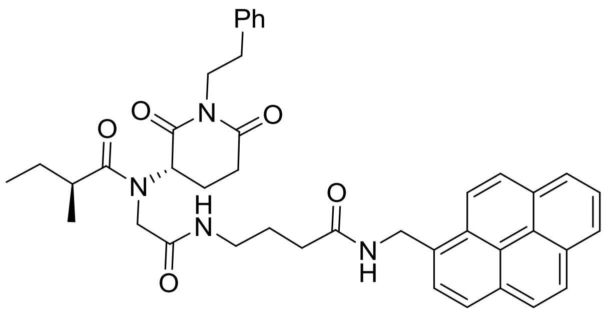

To a solution of **9** (60 mg, 0.13 mmol) in dry dichloromethane (5.0 mL) were added 1-pyrenemethylamine hydrochloride (17 mg, 0.14 mmol), EDCl (26 mg, 0.14 mmol), DMAP (2 mg, 10 mol %) and triethylamine (0.1 mL, 0.39 mmol) at r.t. The reaction mixture was stirred at r.t. for 24 h. The contents were transferred to a separatory funnel and successively washed with 10% v/v HCl (two times, 20 mL), water (once, 10 mL), and brine (once, 10 mL), and dried over anhydrous Na_2SO_4. The solvent was removed under reduced pressure and the crude product purified by gradient silica gel column chromatography with (1:0–8:2) ethyl acetate:methanol as eluent. The obtained material was dissolved in ethyl acetate (10 mL), filtered through a syringe filter (0.45 micron pore size) and evaporated to afford 70 mg of **10** as a light-yellow oil.

Yield: 80%.; $[\alpha]_D^{20}$ −7.0 (*c* 1.0, $CHCl_3$); UV (MeOH) λ_{max} (log ε): 199 (5.49), 233 (5.19), 242 (5.34), 255 (4.72), 265 (4.96), 275 (5.17), 312 (4.68), 326 (4.98), 341 (5.13); ^{1}H NMR δ 0.88 (t, *J* = 7.4 Hz, 3H), 1.10 (d, *J* = 6.8 Hz, 3H), 1.32 (m, 1H), 1.52 (m, 1H), 1.88 (m, 2H), 2.03 (m, 1H), 2.47 (m, 6H), 3.31–3.65 (m, 6H), 3.91–4.61 (m, 3H), 4.90–5.25 (m, 2H), 7.06–7.26 (m, 5H), 7.61 (bs, 1H), 7.86–8.19 (m, 9H), 8.76 (bs, 1H); ^{13}C NMR δ 11.6, 16.9, 20.3, 25.9, 27.3, 30.7, 32.5, 33.5, 36.9, 37.1, 38.2, 41.4, 42.6, 52.8, 60.9, 122.8, 124.4, 124.8, 124.9, 125.4, 125.6, 126.1, 126.3, 127.3, 128.2, 128.6,

128.8, 128.9, 130.2, 130.6, 131.2, 131.3, 138.4, 169.4, 169.9, 172.1, 172.9; HRMS *m*/*z* cald for $C_{41}H_{44}N_4NaO_5$, 695.3209; found, 695.3204.

References

1 Kaldor, S. W.; Hammond, M.; Dressman, B. A.; Labus, J. M.; Chadwell, F. W.; Kline, A. D.; Heinz, B. A. *Bioorg. Med. Chem. Lett.* **1995**, *5*, 2021-2026.

2 Lee, J.; Son, K.; Kim, M.; Jung, G.; Choi, J.; Lee, E. S.; Park, M. *Arch. Pharm. Res.* **1999**, *22*, 491-495.

3 Sen, S. E.; Roach, S. L. *Synthesis* **1995**, 756-758.

4 Silva, L. L.; Joussef, A. C. *J. Nat. Prod.* **2011**, *74*, 1531-1534.

5 The formation of a colored pigment during the hydrogenation of the intermediate **4** has already been reported. For more details see: Sondheimer, E.; Holley, R. W. *J. Am. Chem. Soc.* **1957**, *79*, 3767-3770.

6 After addition of formaldehyde initially a methanolic suspension is formed, that after approximately 2 h becomes clear as the oligomeric starting material is consumed. It indicates that imine formation is completed as can be easily confirmed by ESI-MS. It is important not to add the carboxylic acid and isonitrile components to a solution still containing unreacted formaldehyde as otherwise the yields of desired products decrease and the competing Passerini reaction is observed.

7 Evans, R.L.; Irreverre, F. *J. Org. Chem.* **1959**, *24*, 863-864.

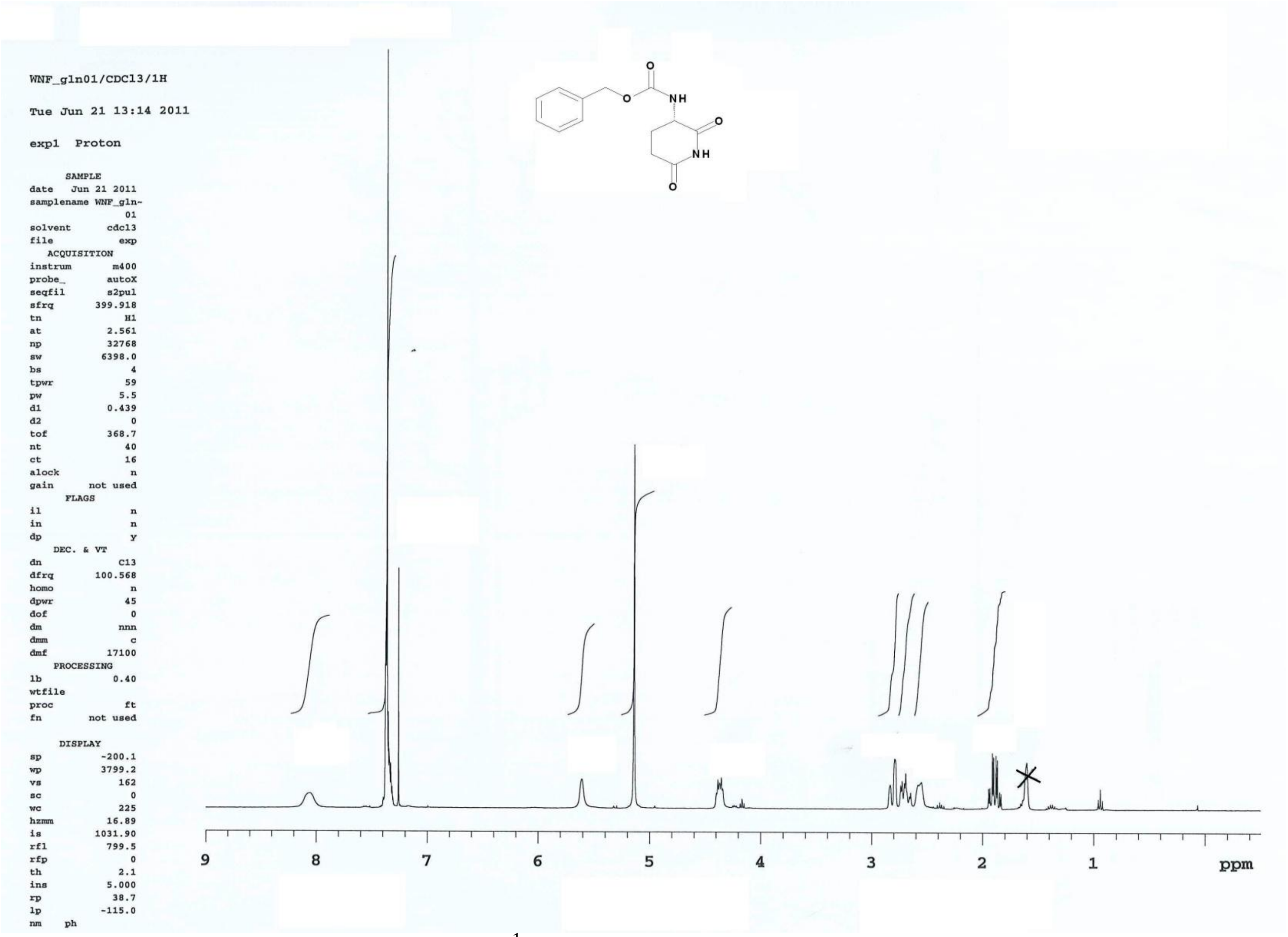

Figure S1: ^{1}H NMR spectrum of compound **3**.

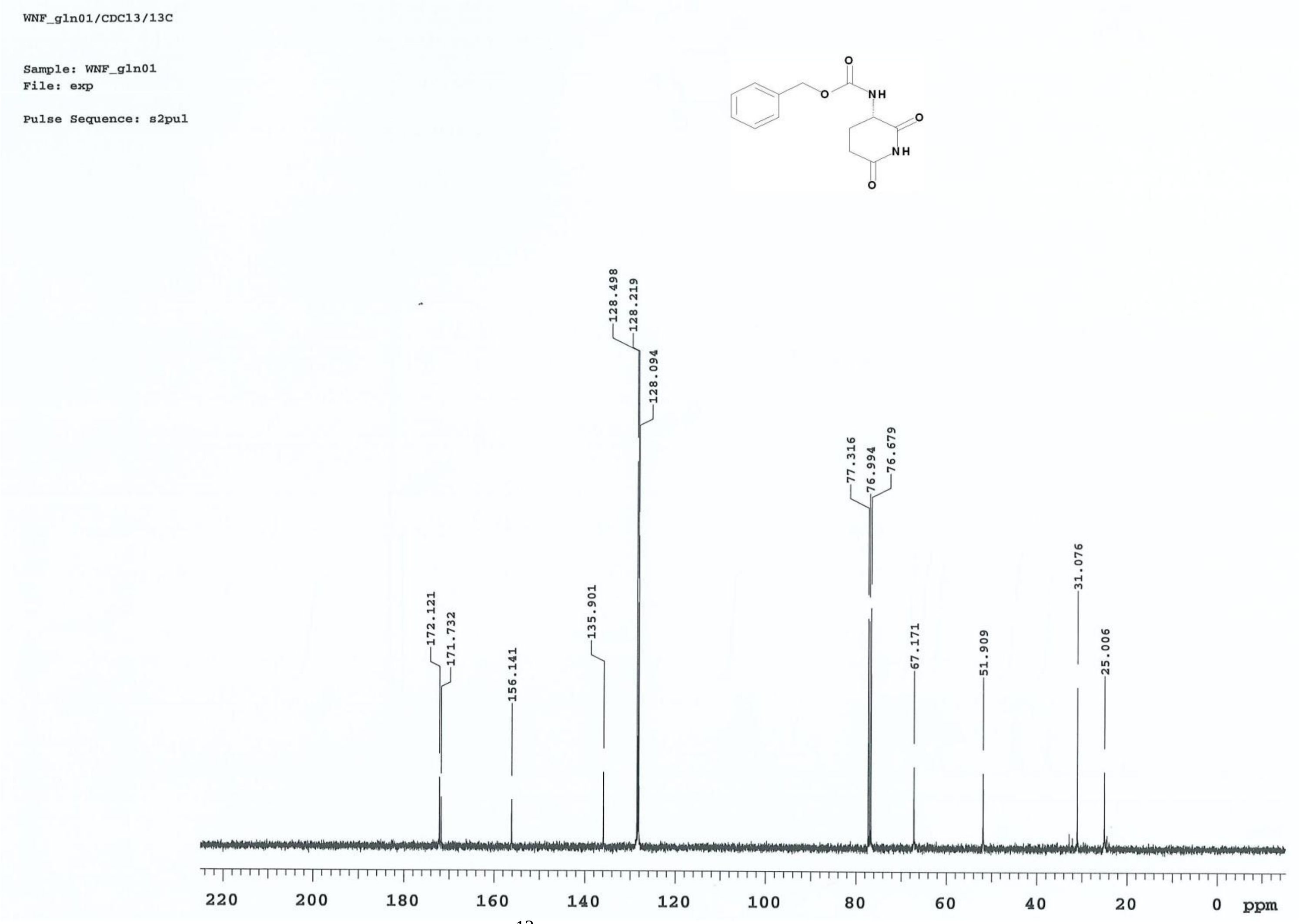

Figure S2: ^{13}C NMR spectrum of compound **3**.

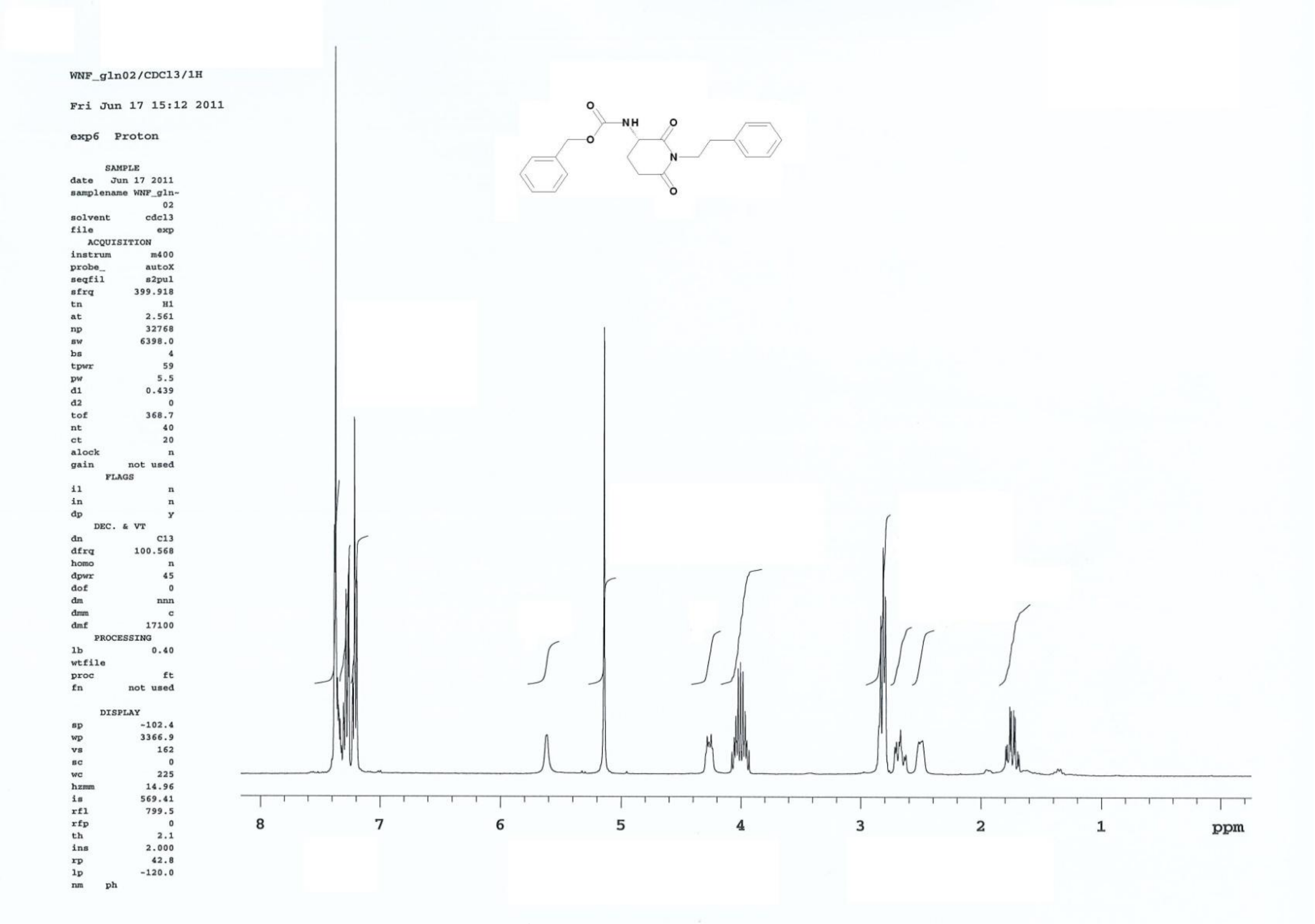

Figure S3: ^{1}H NMR spectrum of compound **4**.

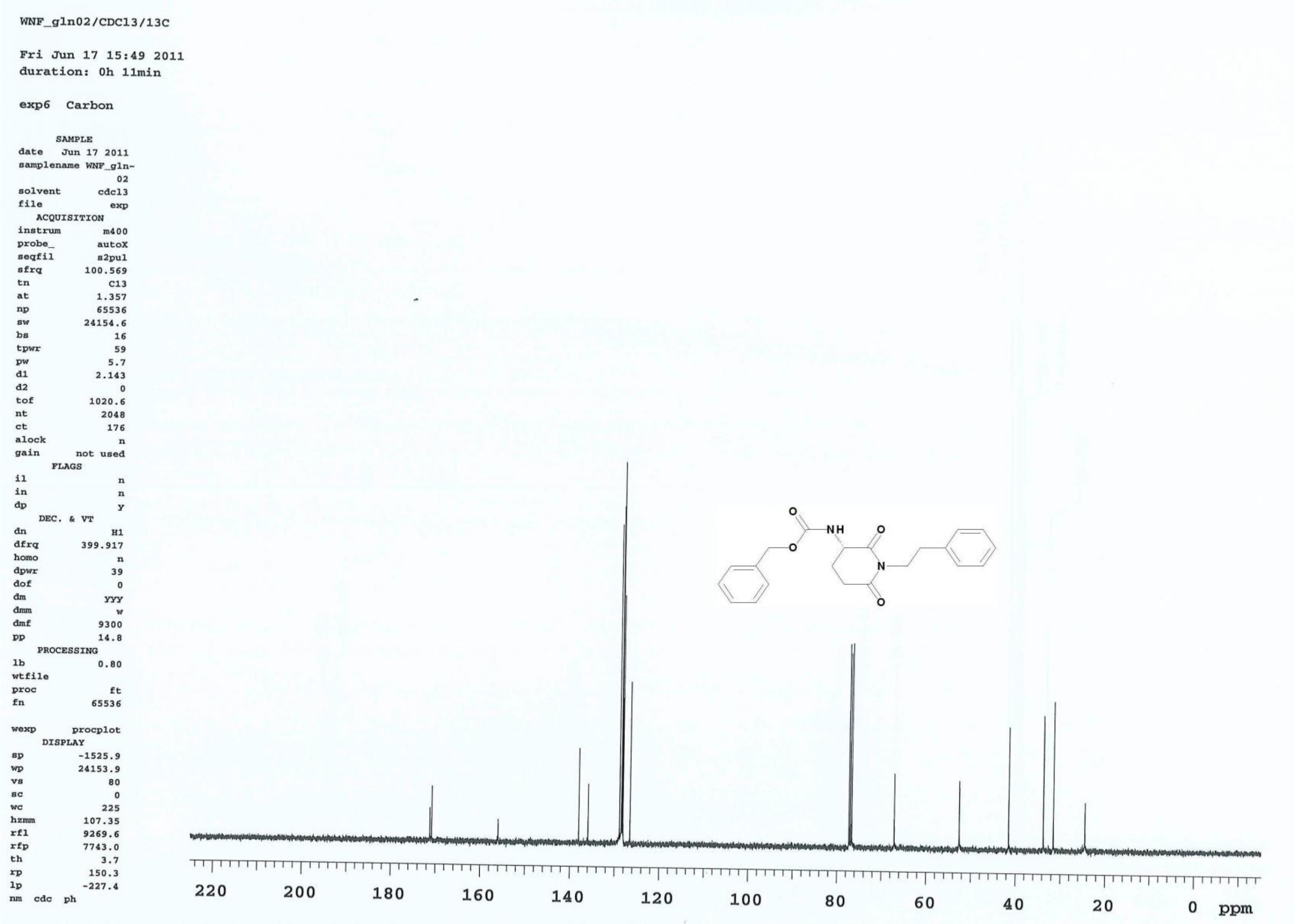

Figure S4: ^{13}C NMR spectrum of compound **4**.

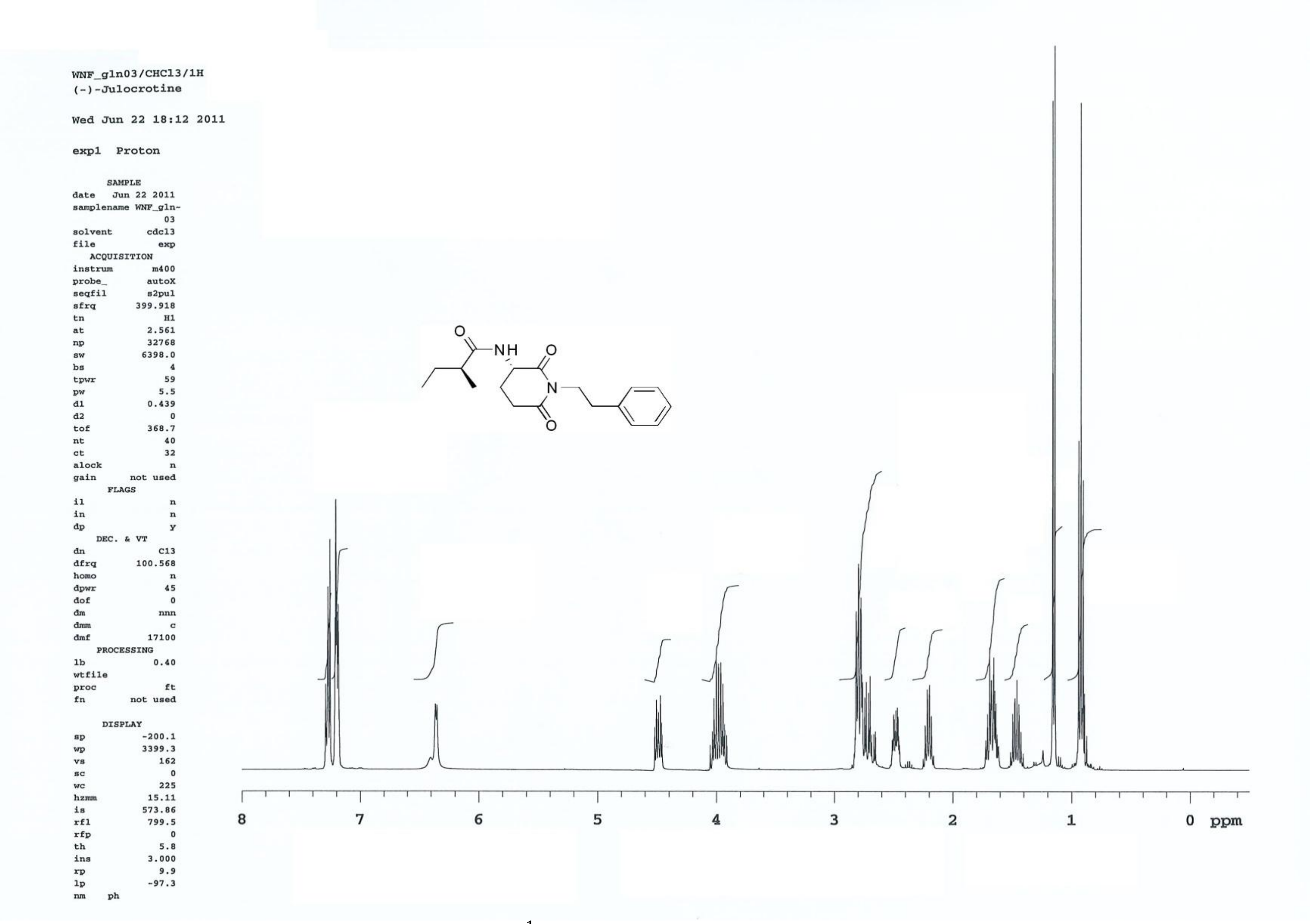

Figure S5: ^{1}H NMR spectrum of compound **1**.

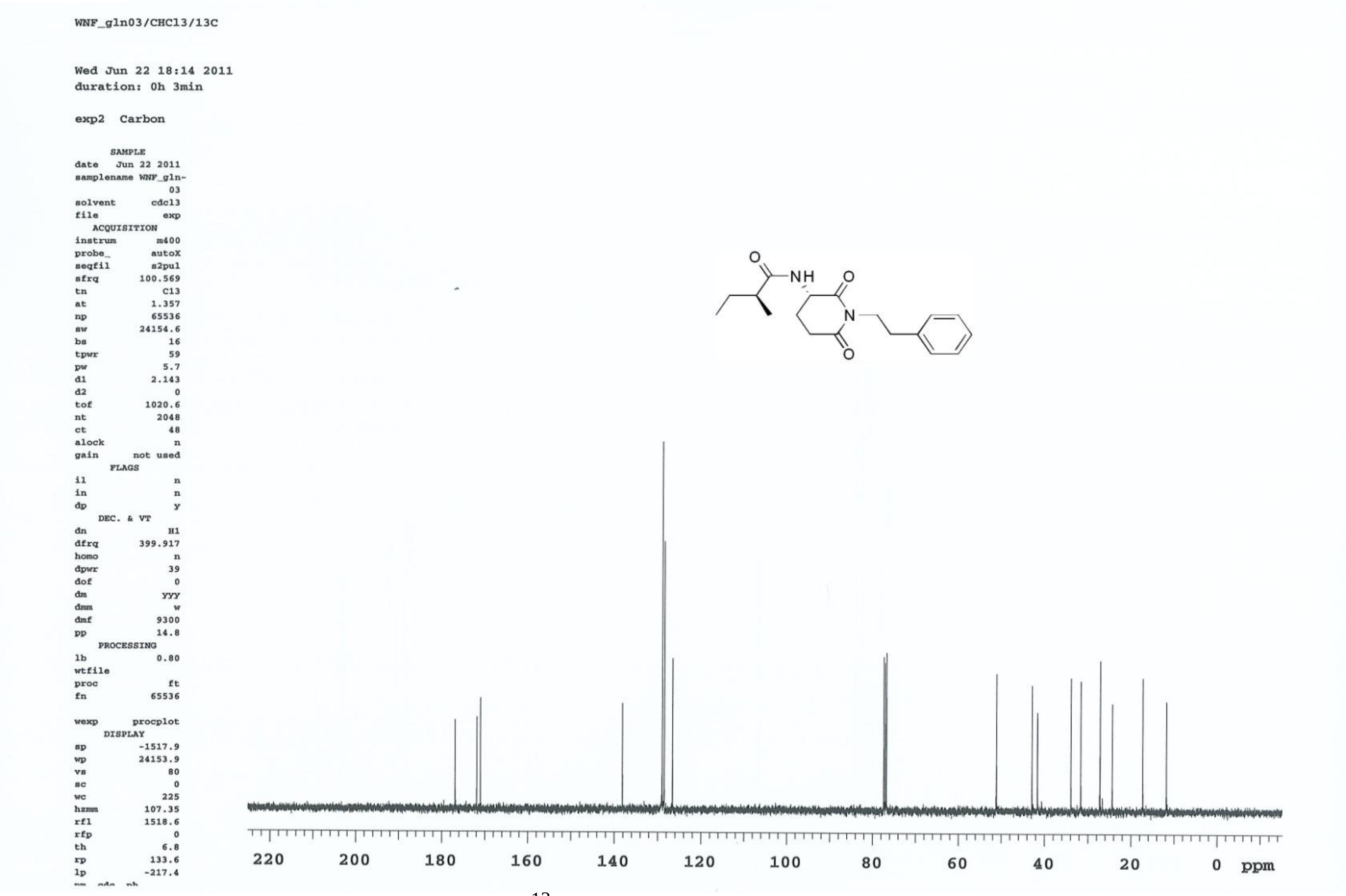

Figure S6: ^{13}C NMR spectrum of compound **1**.

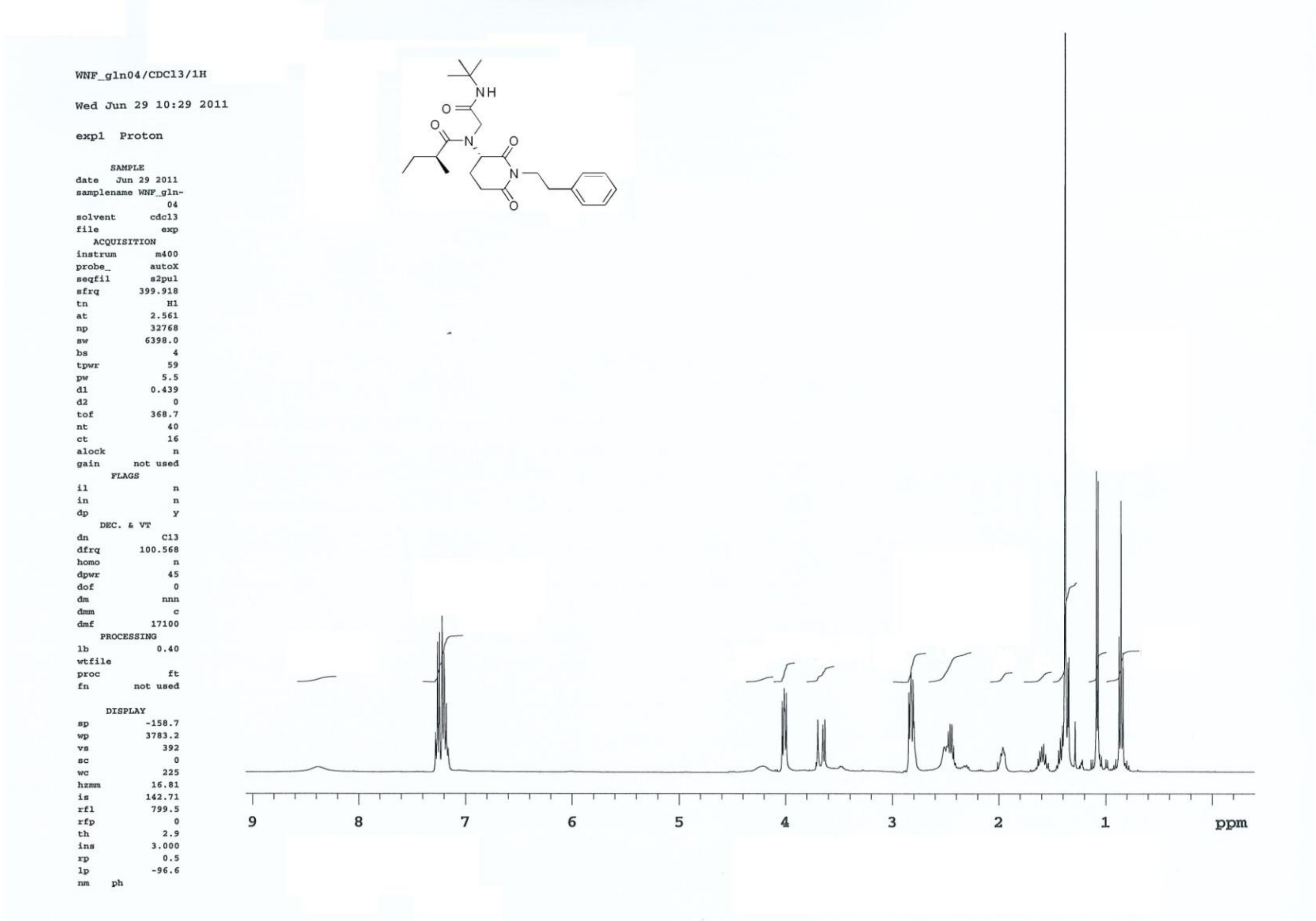

Figure S7: ^{1}H NMR spectrum of compound **6a**.

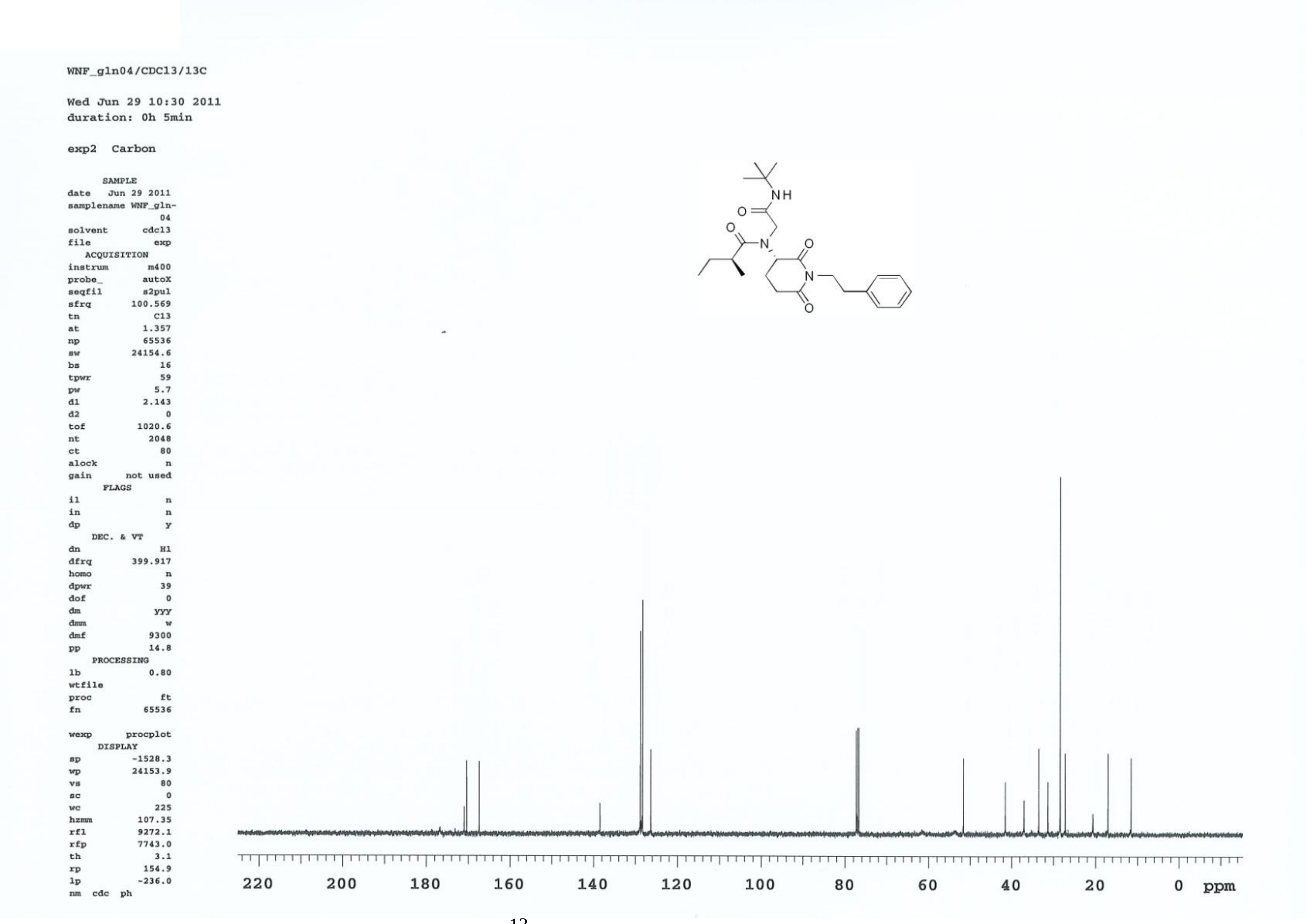

Figure S8: ^{13}C NMR spectrum of compound **6a**.

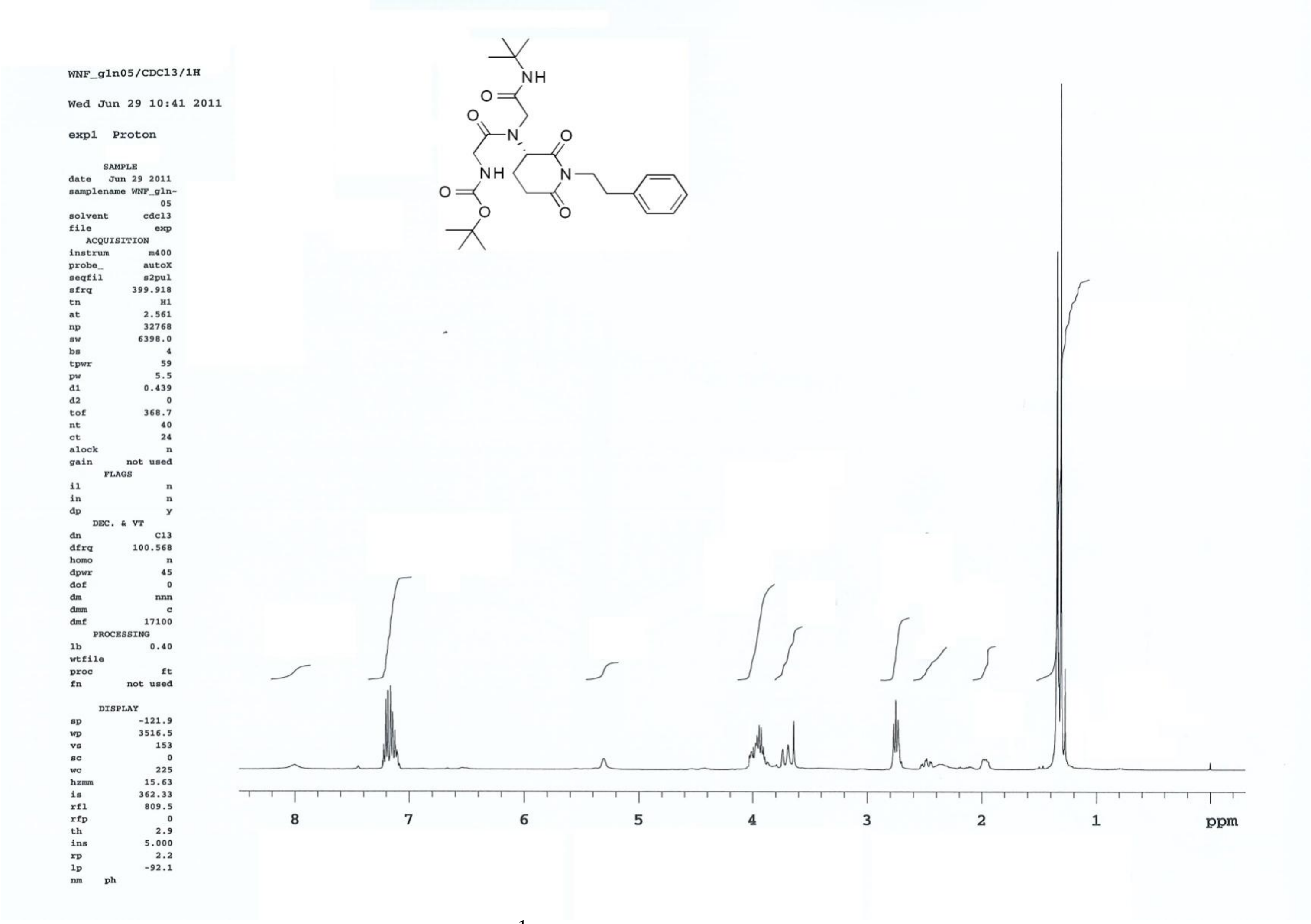

Figure S9: ^{1}H NMR spectrum of compound **6b**.

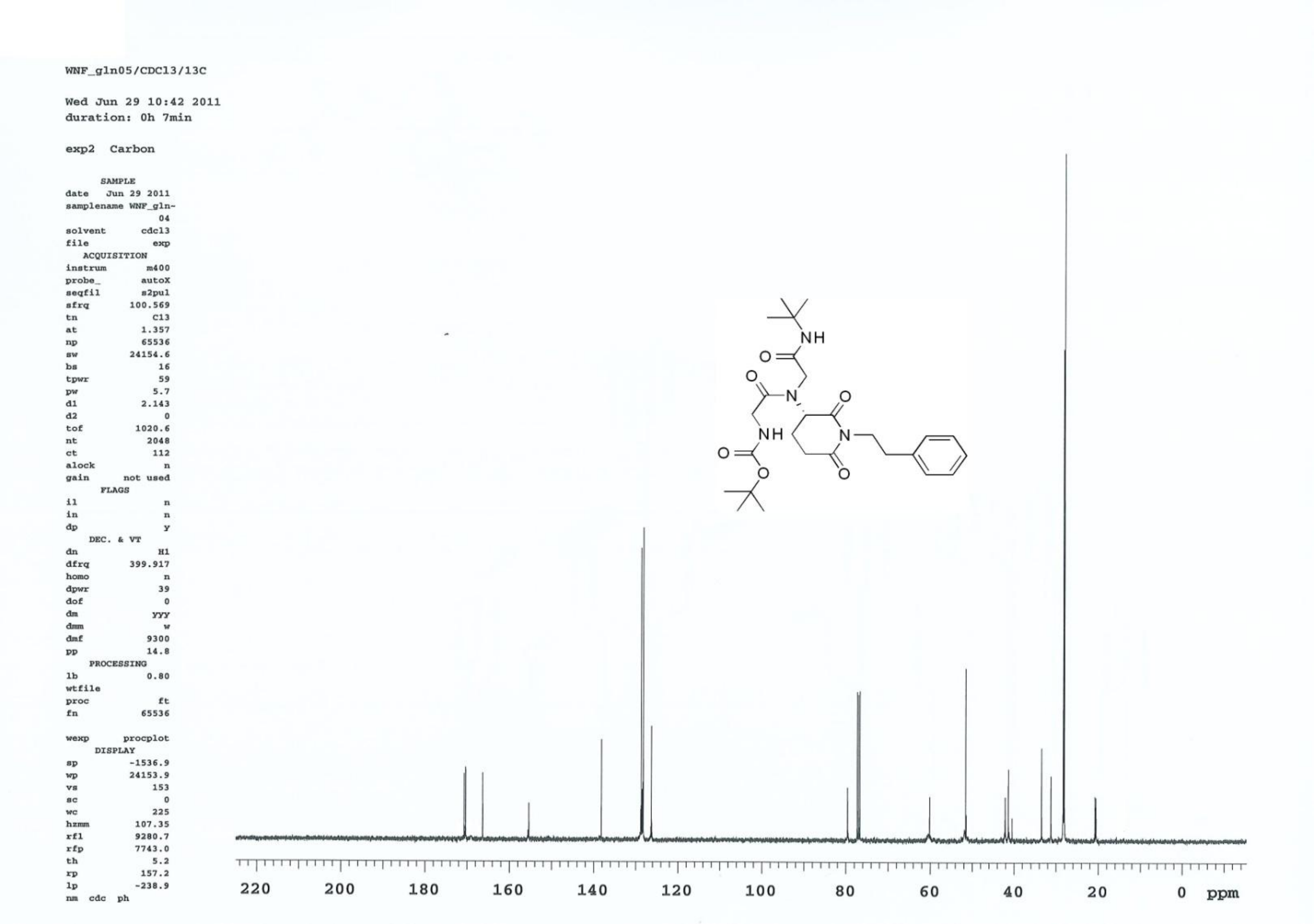

Figure S10: ^{13}C NMR spectrum of compound **6b**.

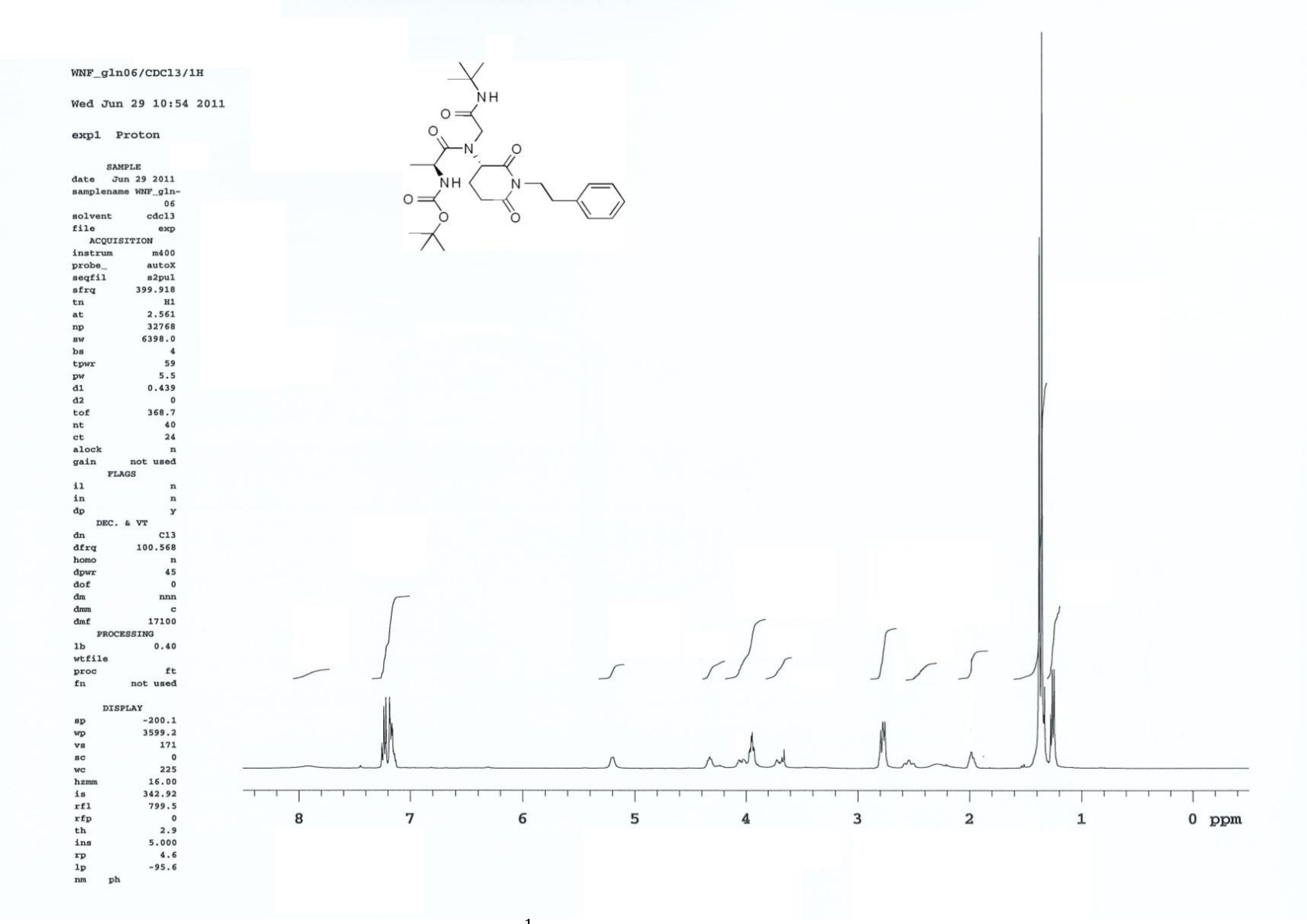

Figure S11: ^{1}H NMR spectrum of compound **6c**.

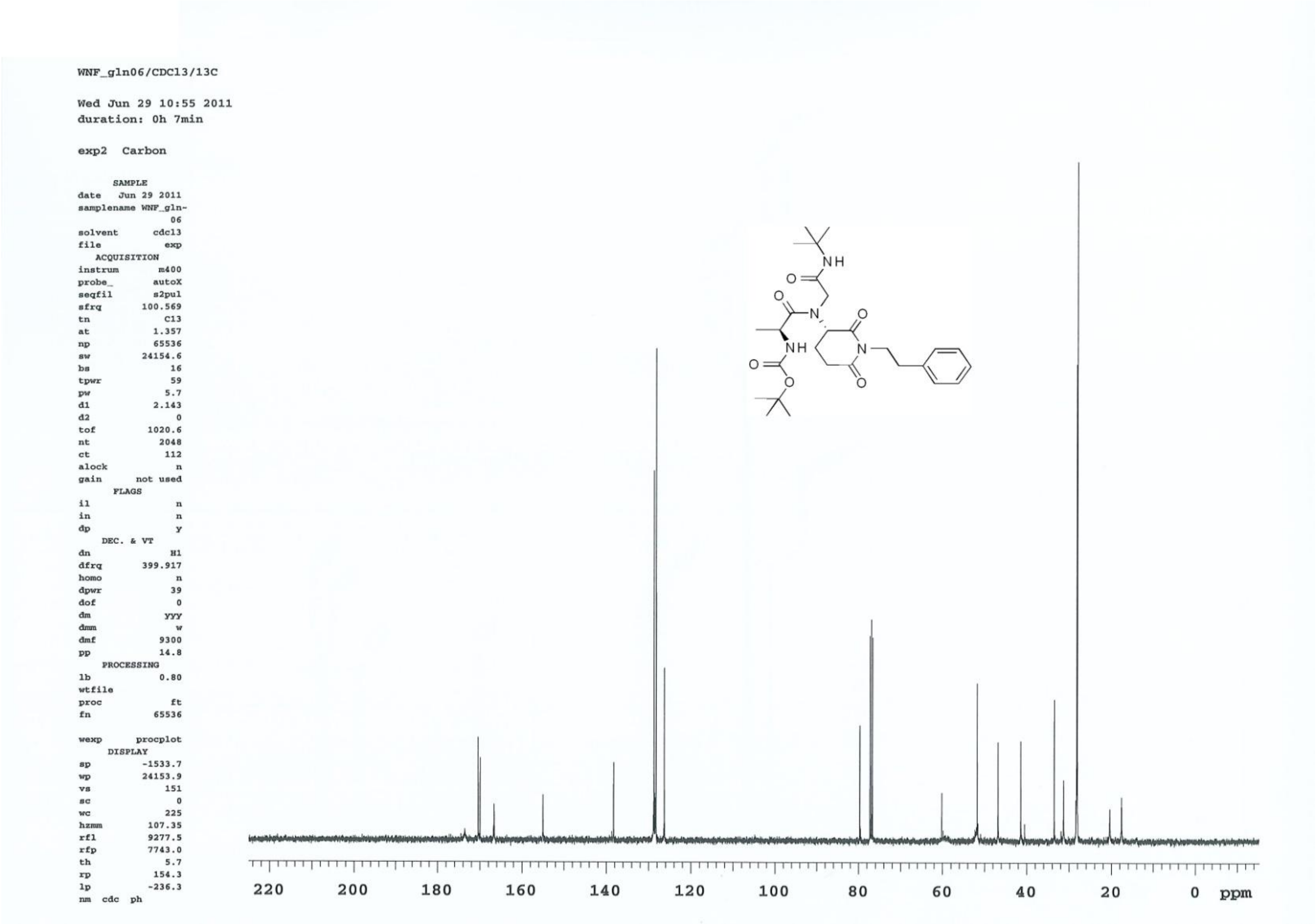

Figure S12: ^{13}C NMR spectrum of compound **6c**.

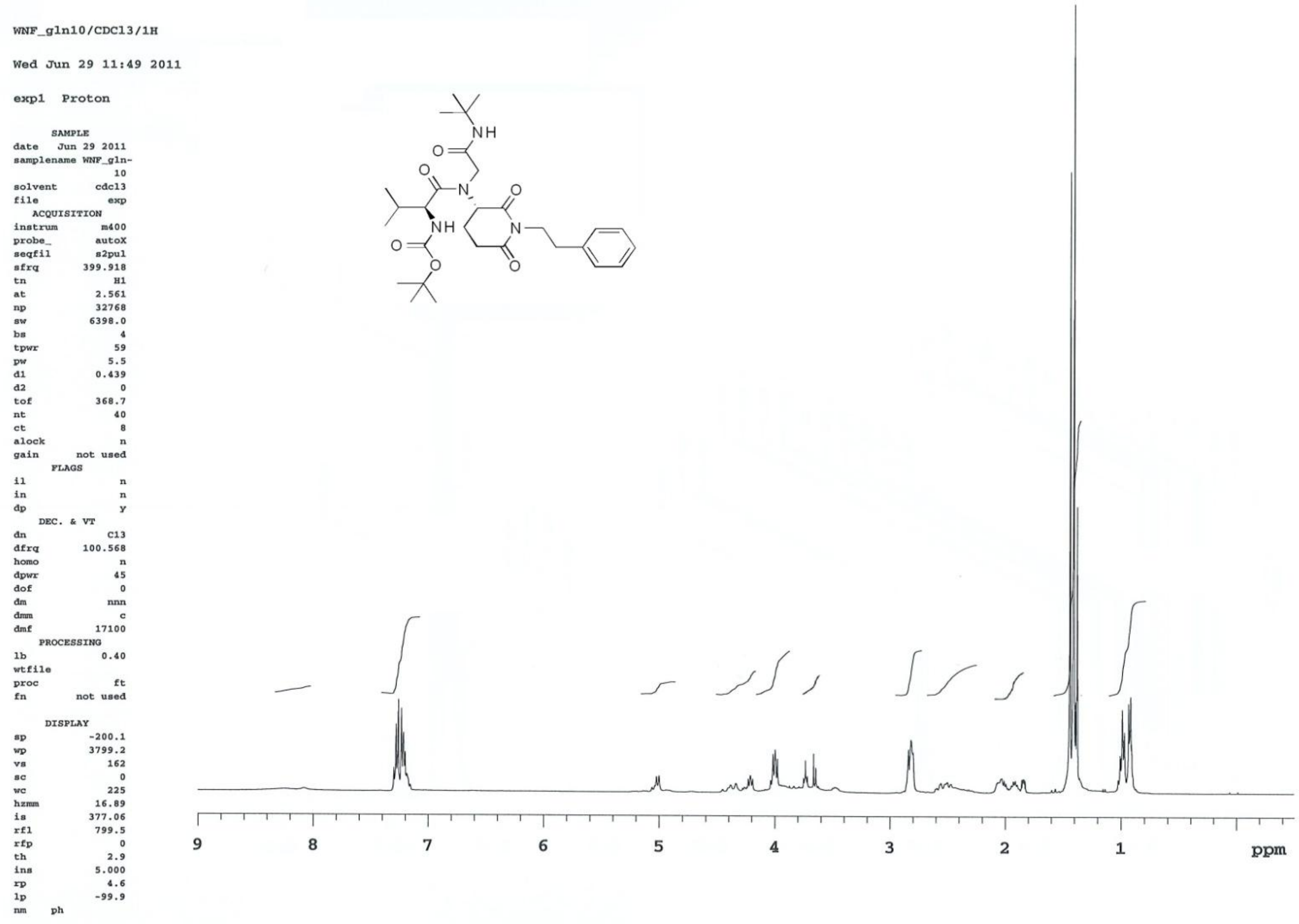

Figure S13: ^{1}H NMR spectrum of compound **6d**.

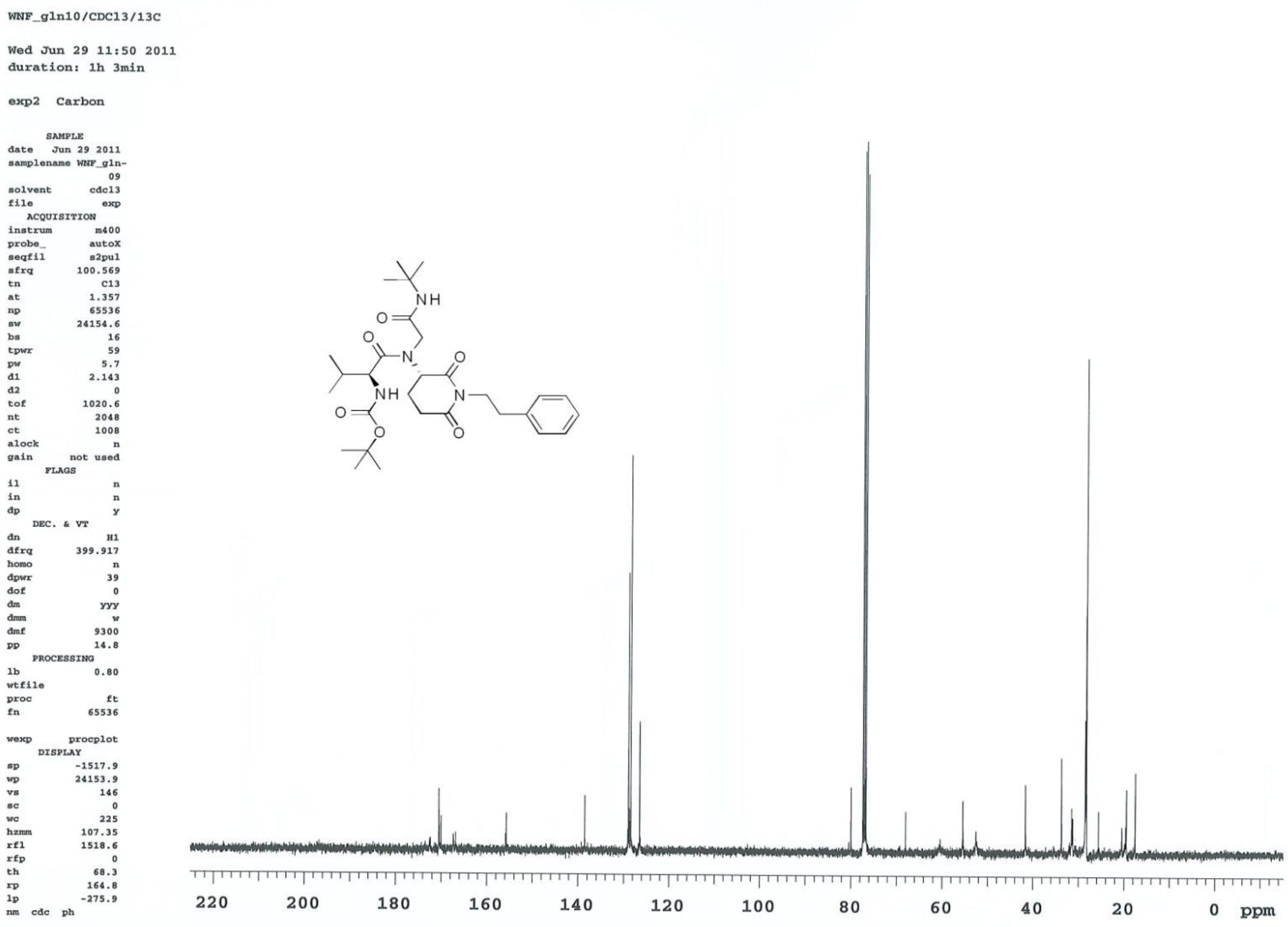

Figure S14: ^{13}C NMR spectrum of compound **6d**.

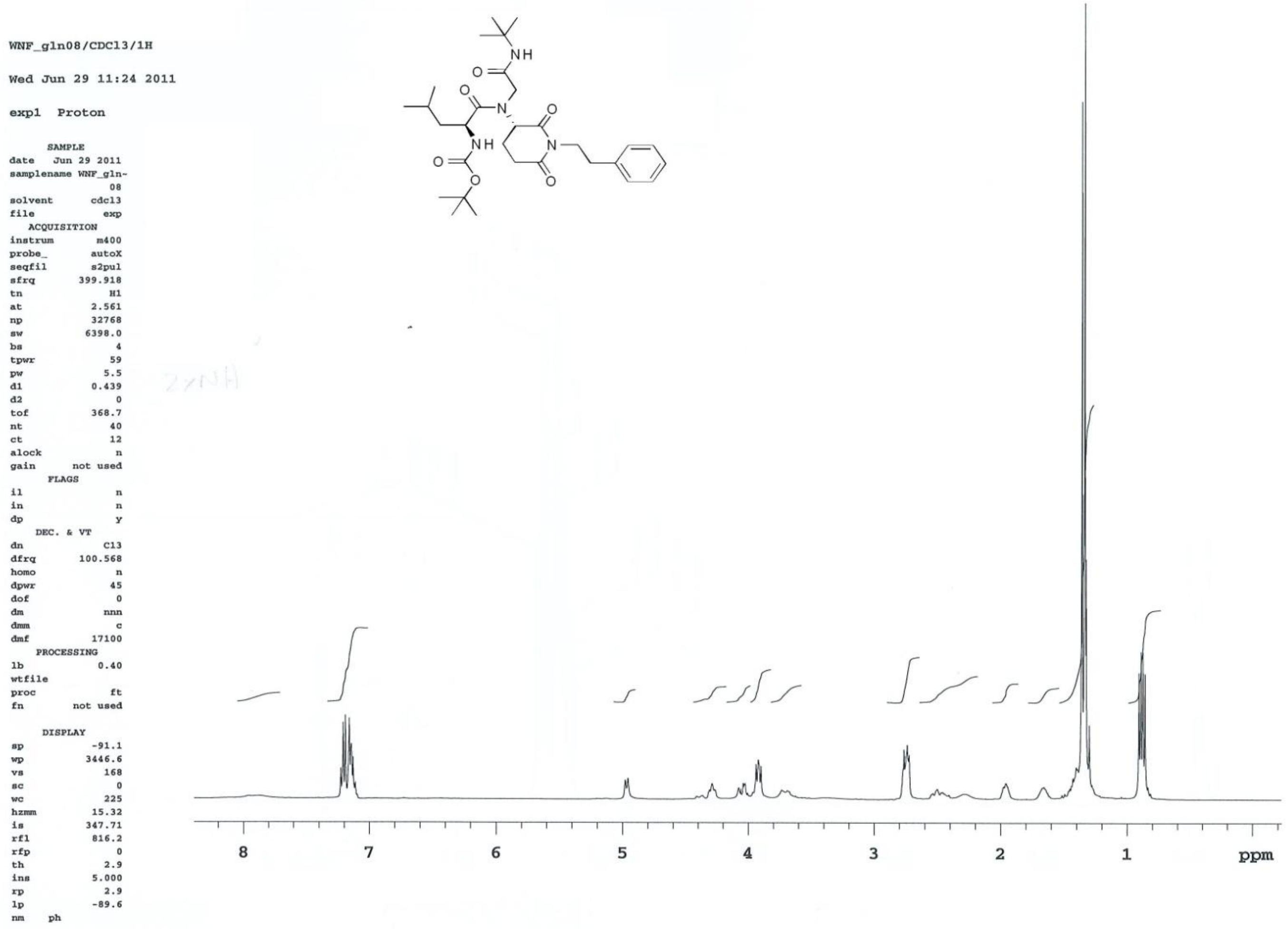

Figure S15: ^{1}H NMR spectrum of compound **6e**.

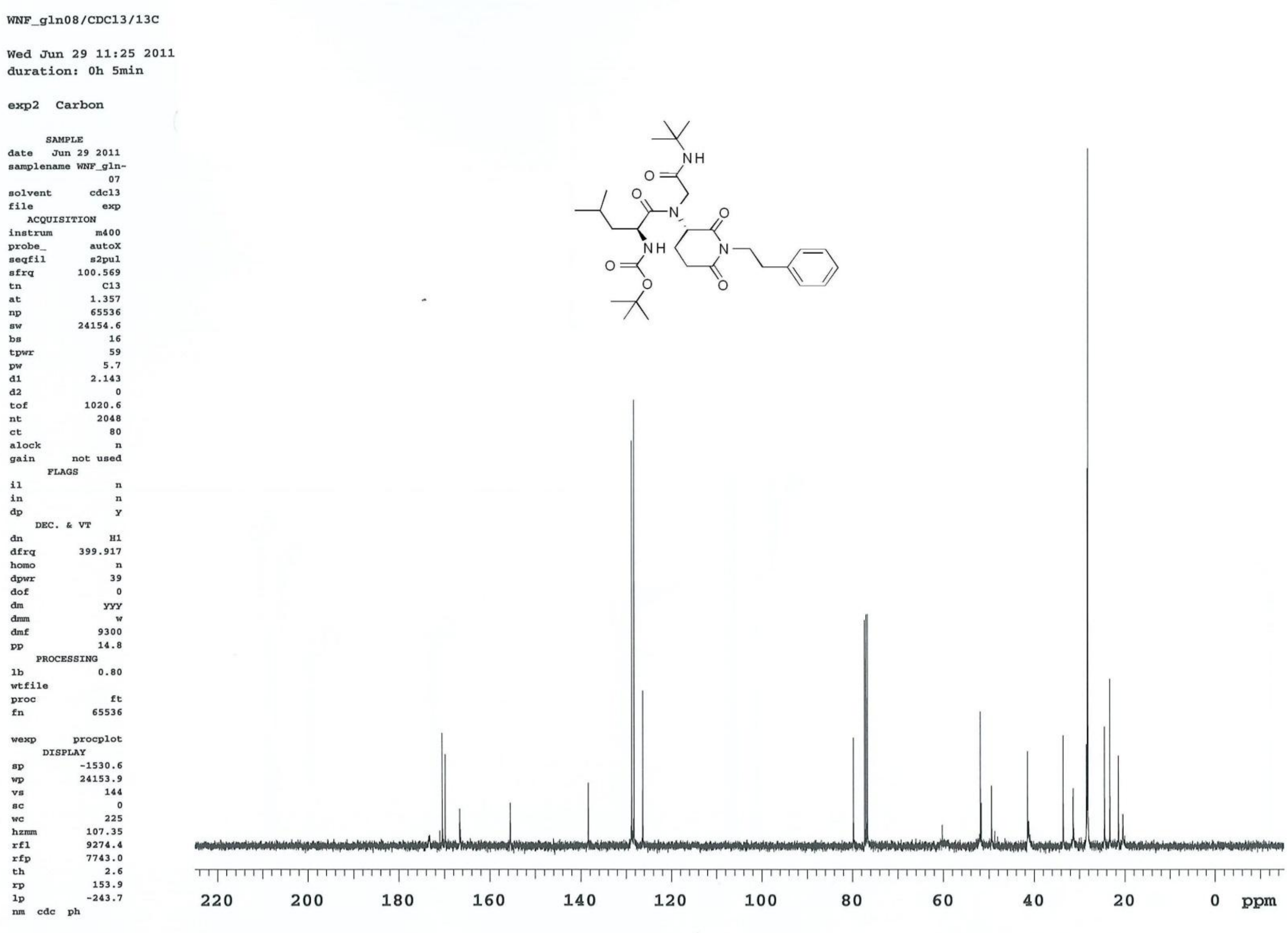

Figure S16: ^{13}C NMR spectrum of compound **6e**.

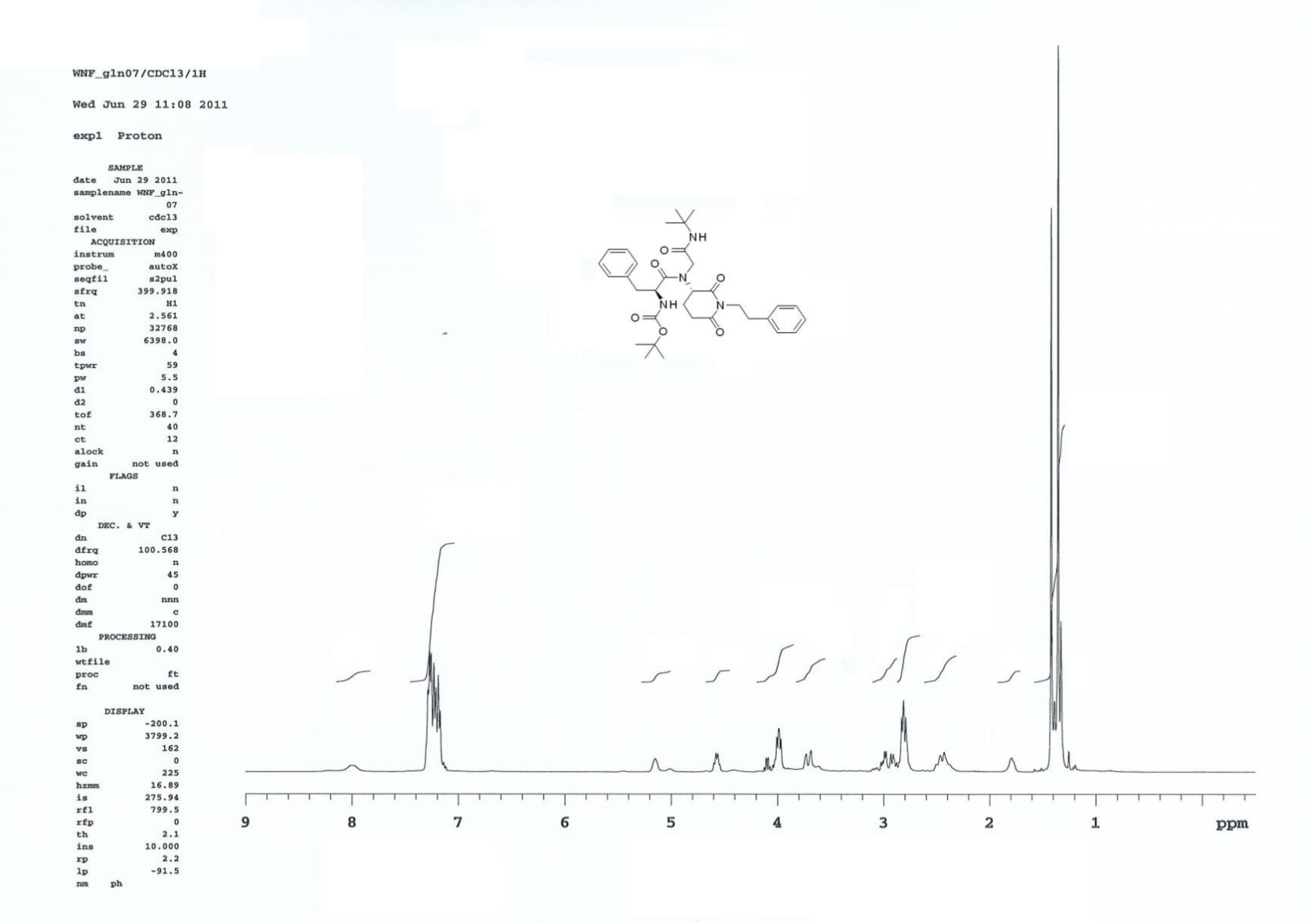

Figure S17: ^{1}H NMR spectrum of compound **6f**.

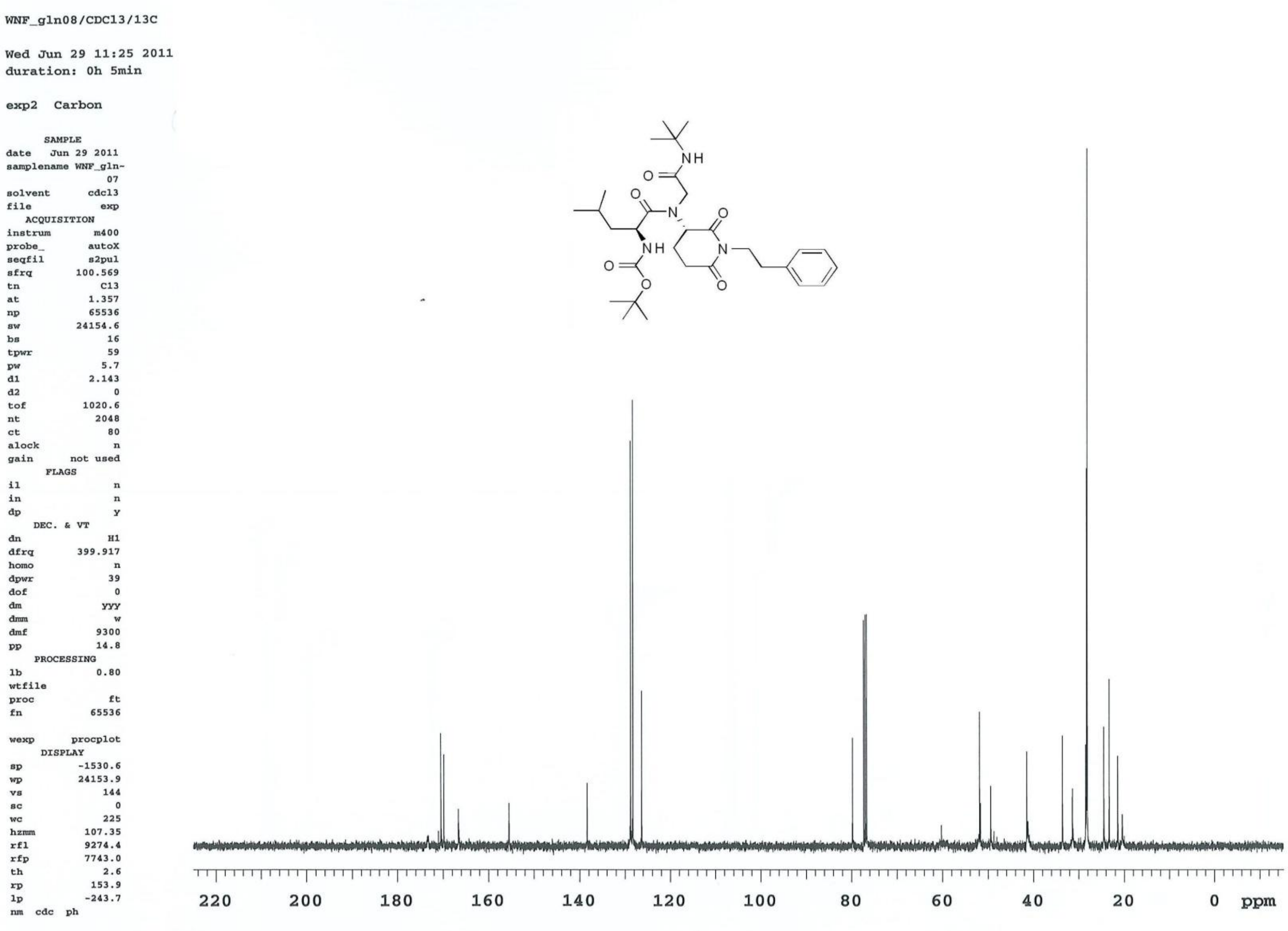

Figure S18: ^{13}C NMR spectrum of compound **6f**.

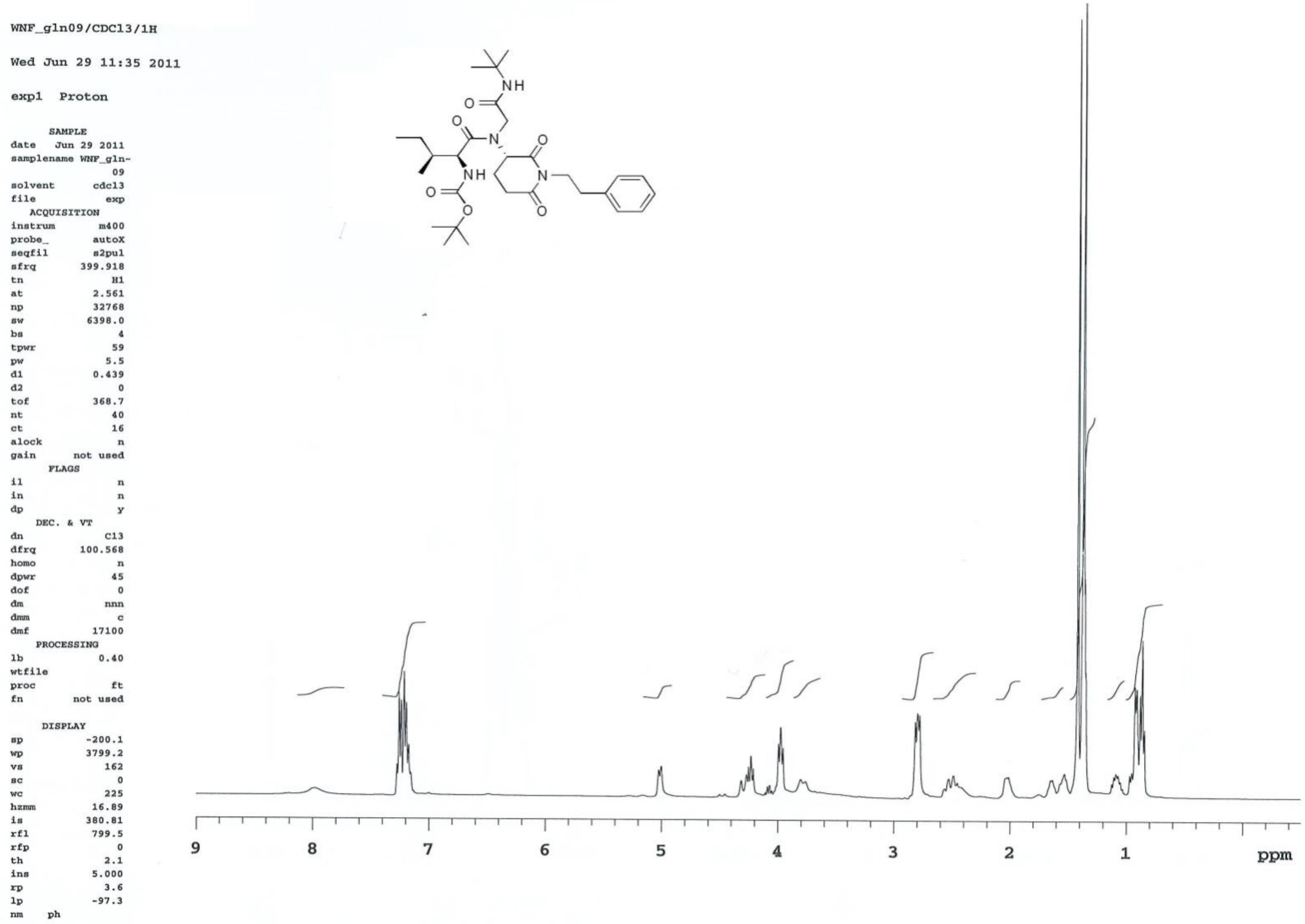

Figure S19: ^{1}H NMR spectrum of compound **6g**.

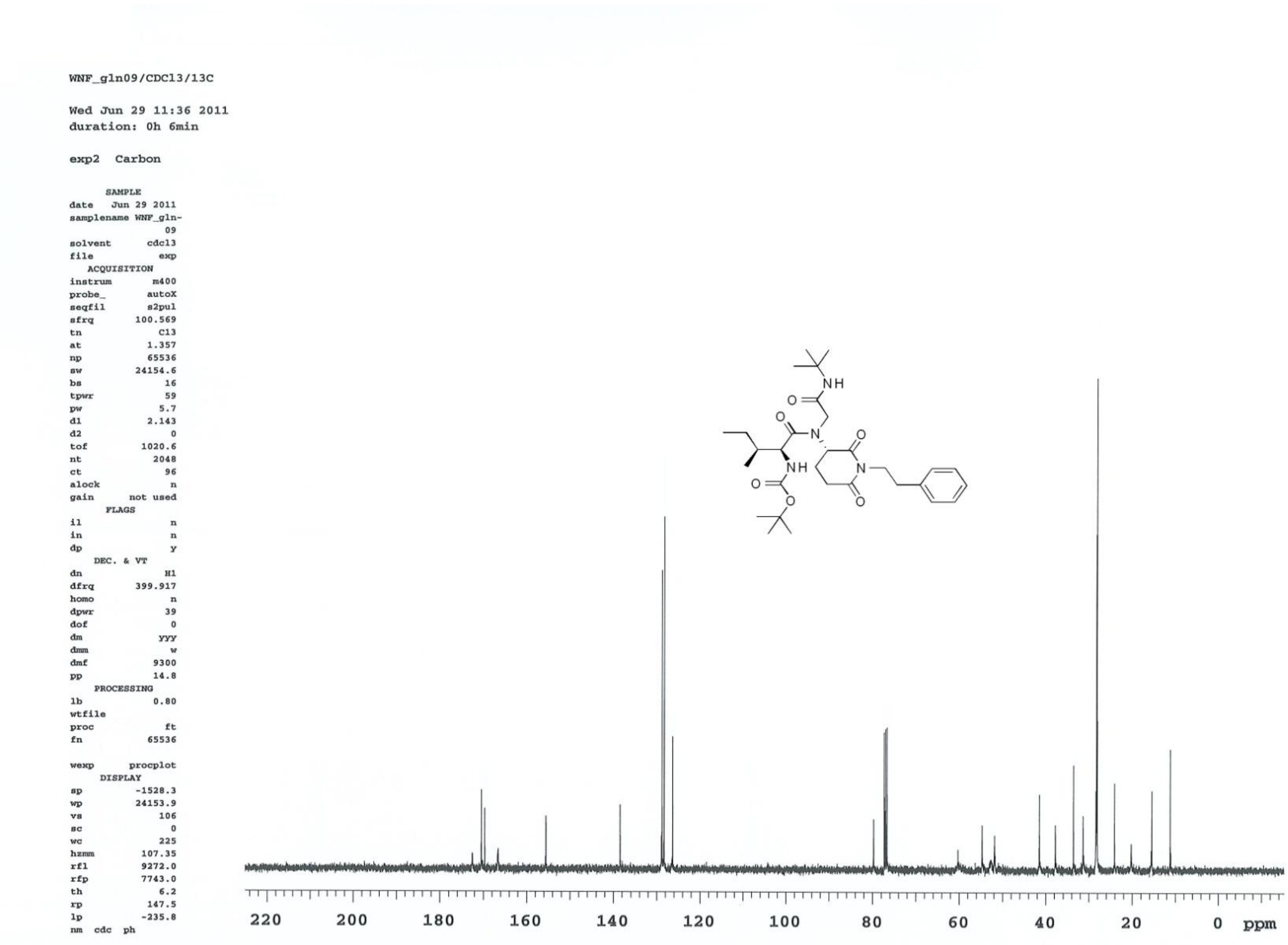

Figure S19: ^{13}C NMR spectrum of compound **6g**.

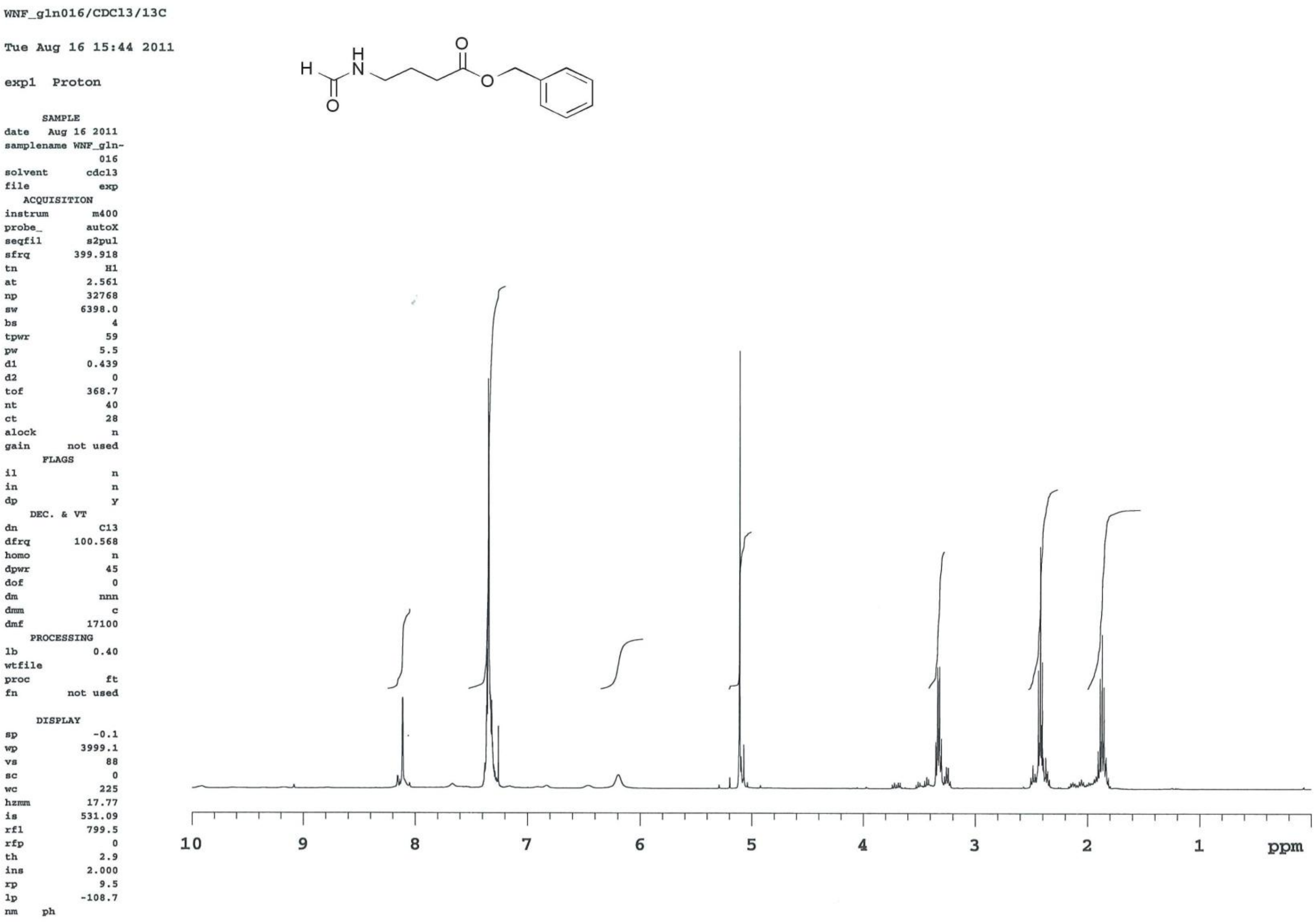

Figure S20: ^{1}H NMR spectrum of benzyl 4-formamidobutanoate.

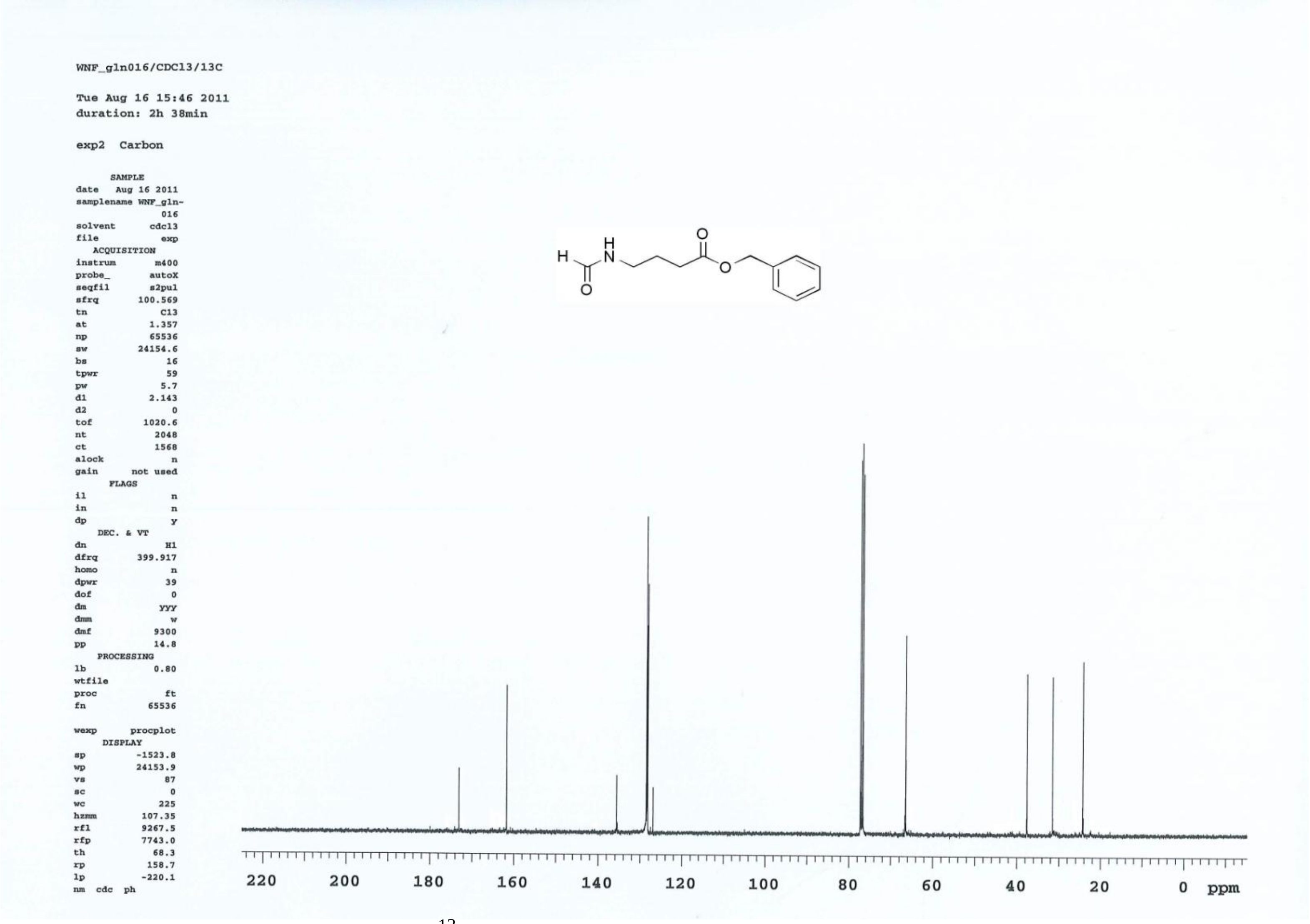

Figure S21: ^{13}C NMR spectrum of benzyl 4-formamidobutanoate.

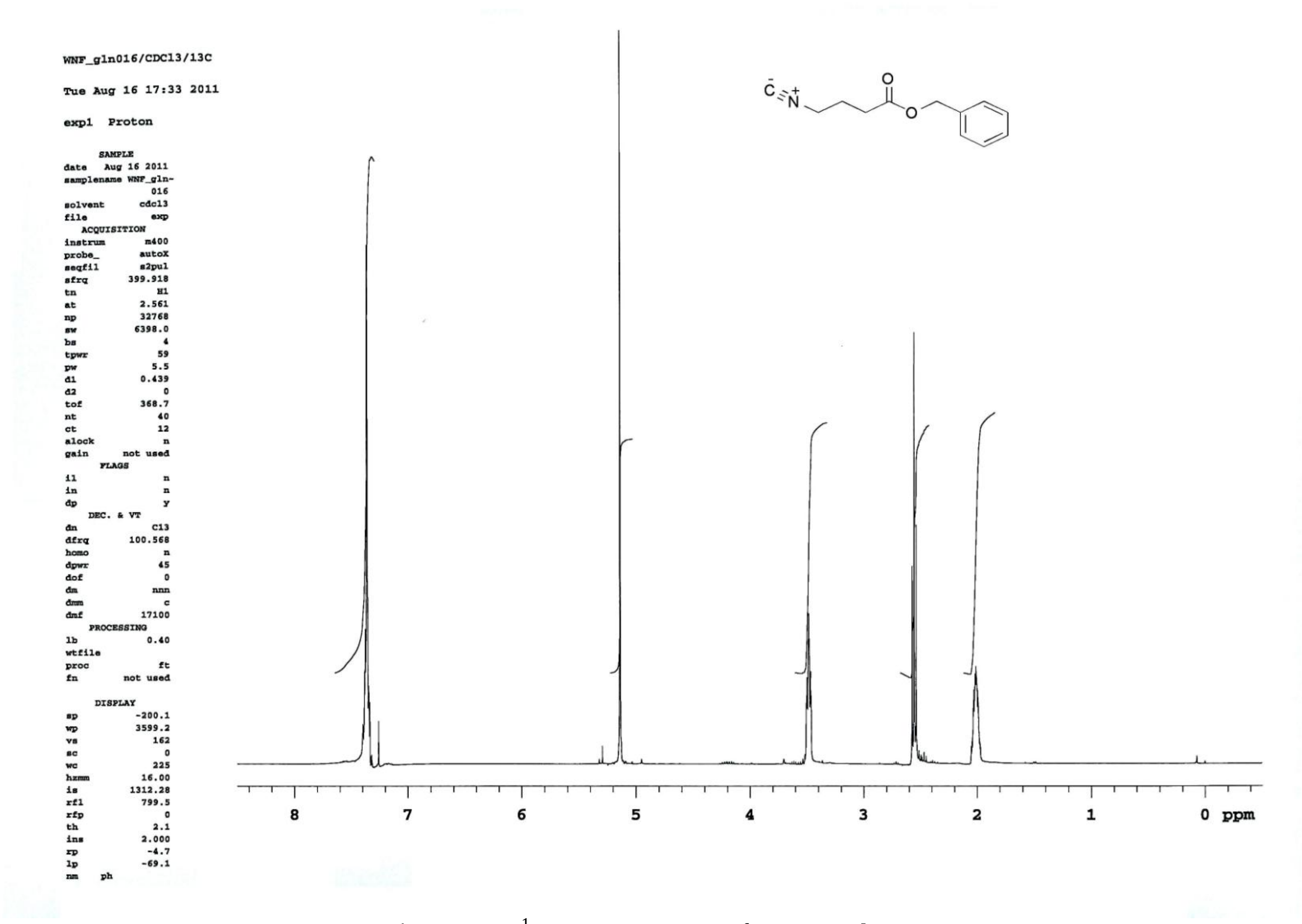

Figure S22: ^{1}H NMR spectrum of compound **7**.

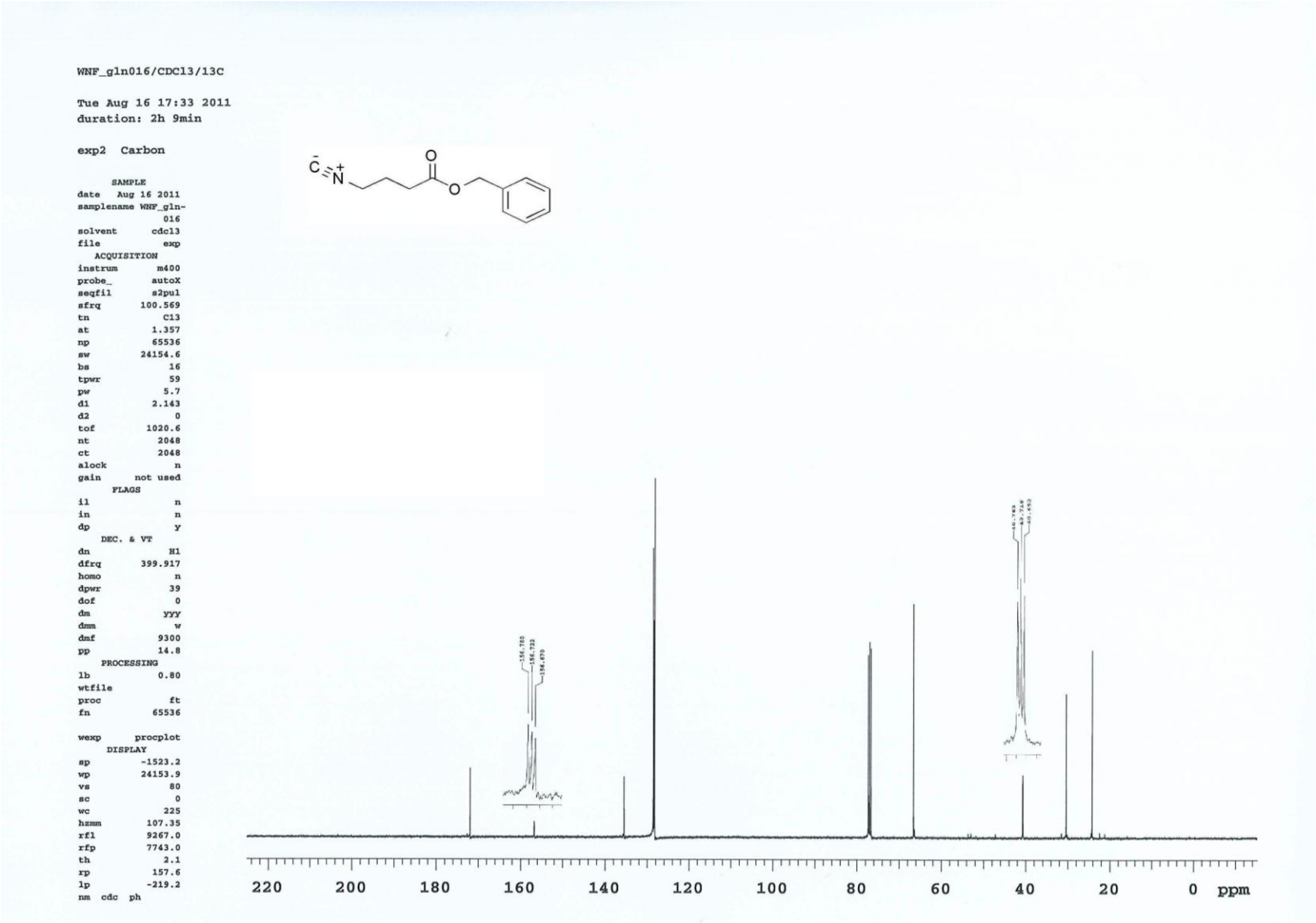

Figure S23: ^{13}C NMR spectrum of compound **7**.

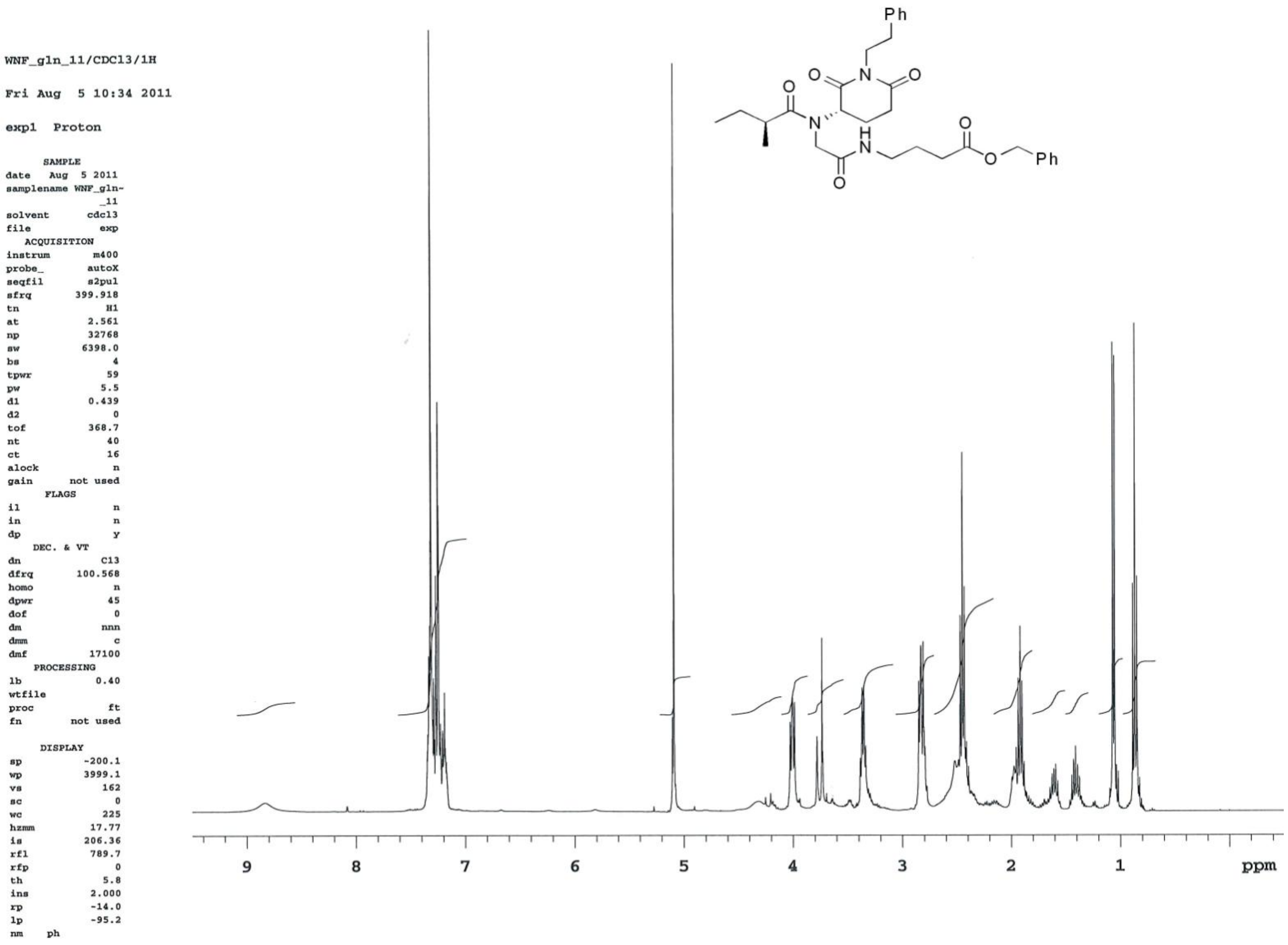

Figure S24: ^{1}H NMR spectrum of compound **8**.

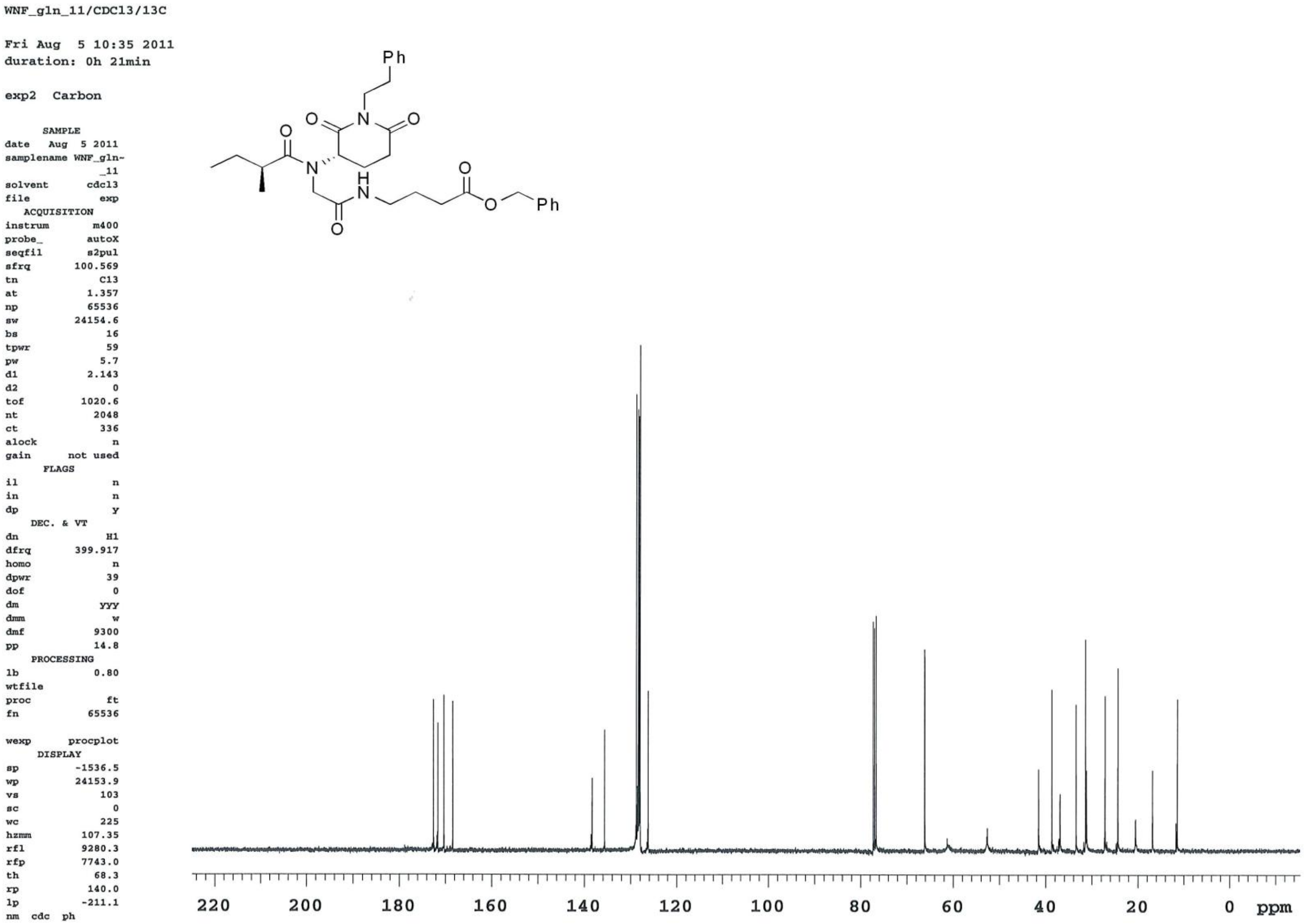

Figure S25: ^{13}C NMR spectrum of compound **8**.

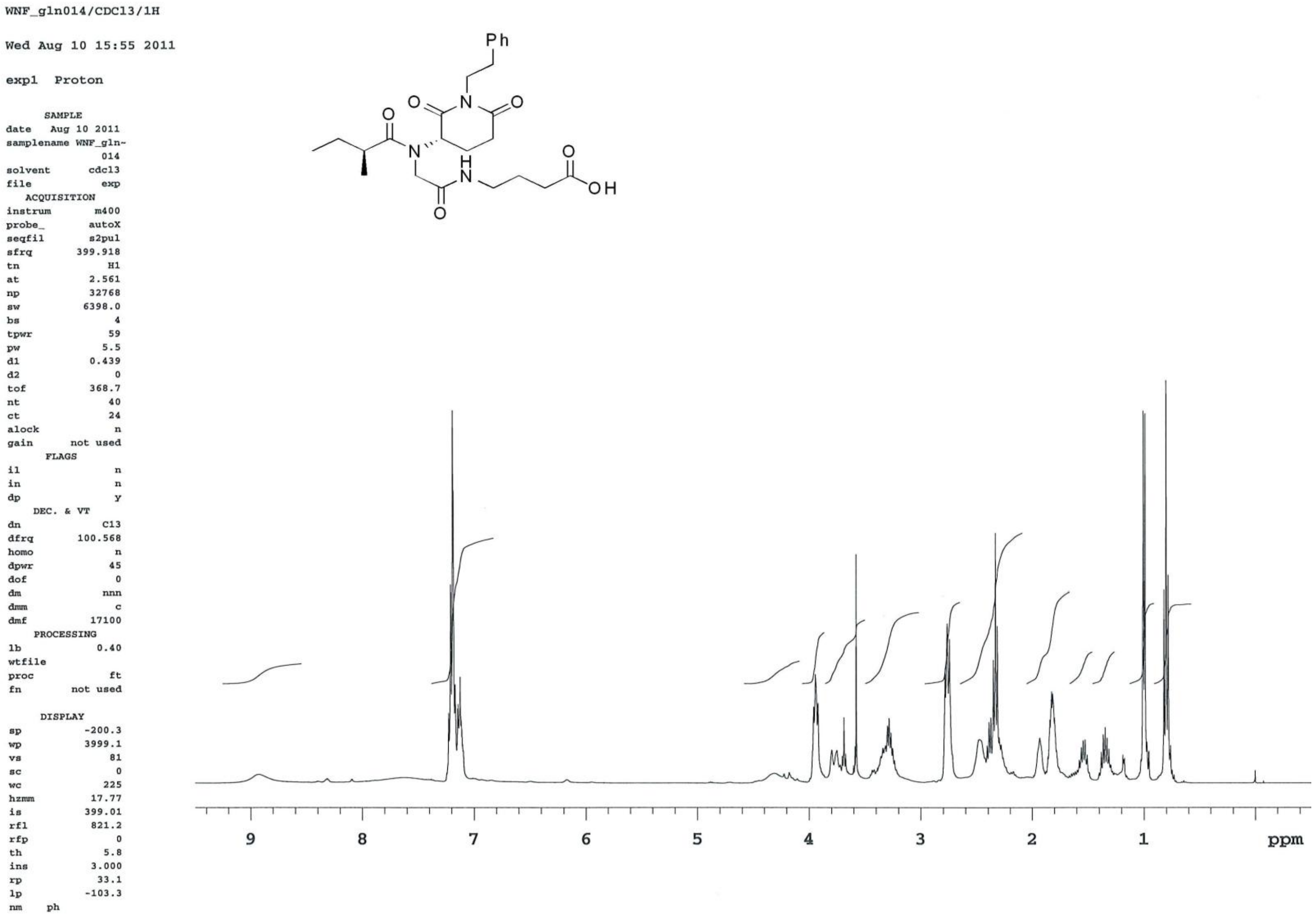

Figure S26: ^{1}H NMR spectrum of compound **9**.

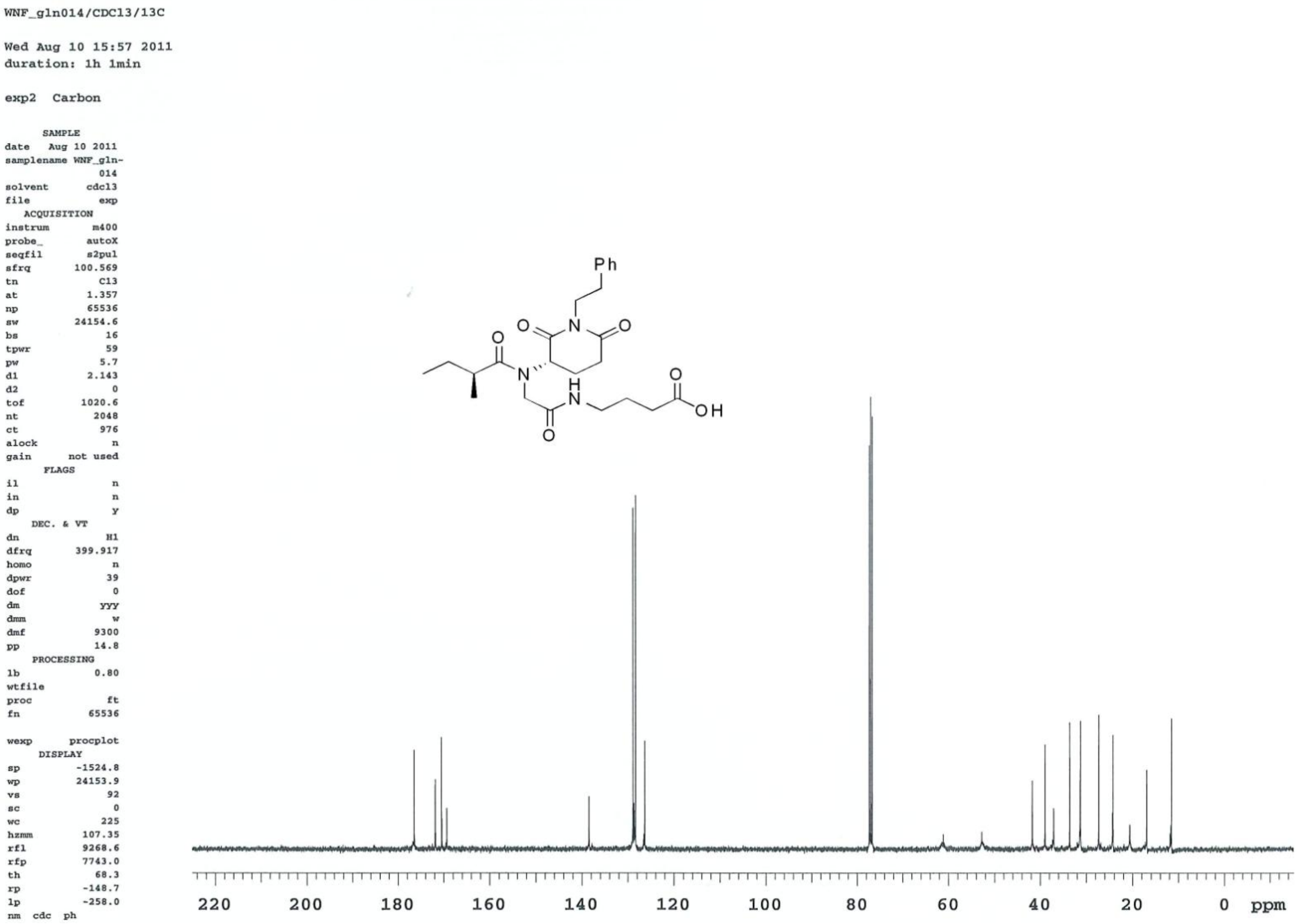

Figure S27: ^{13}C NMR spectrum of compound **9**.

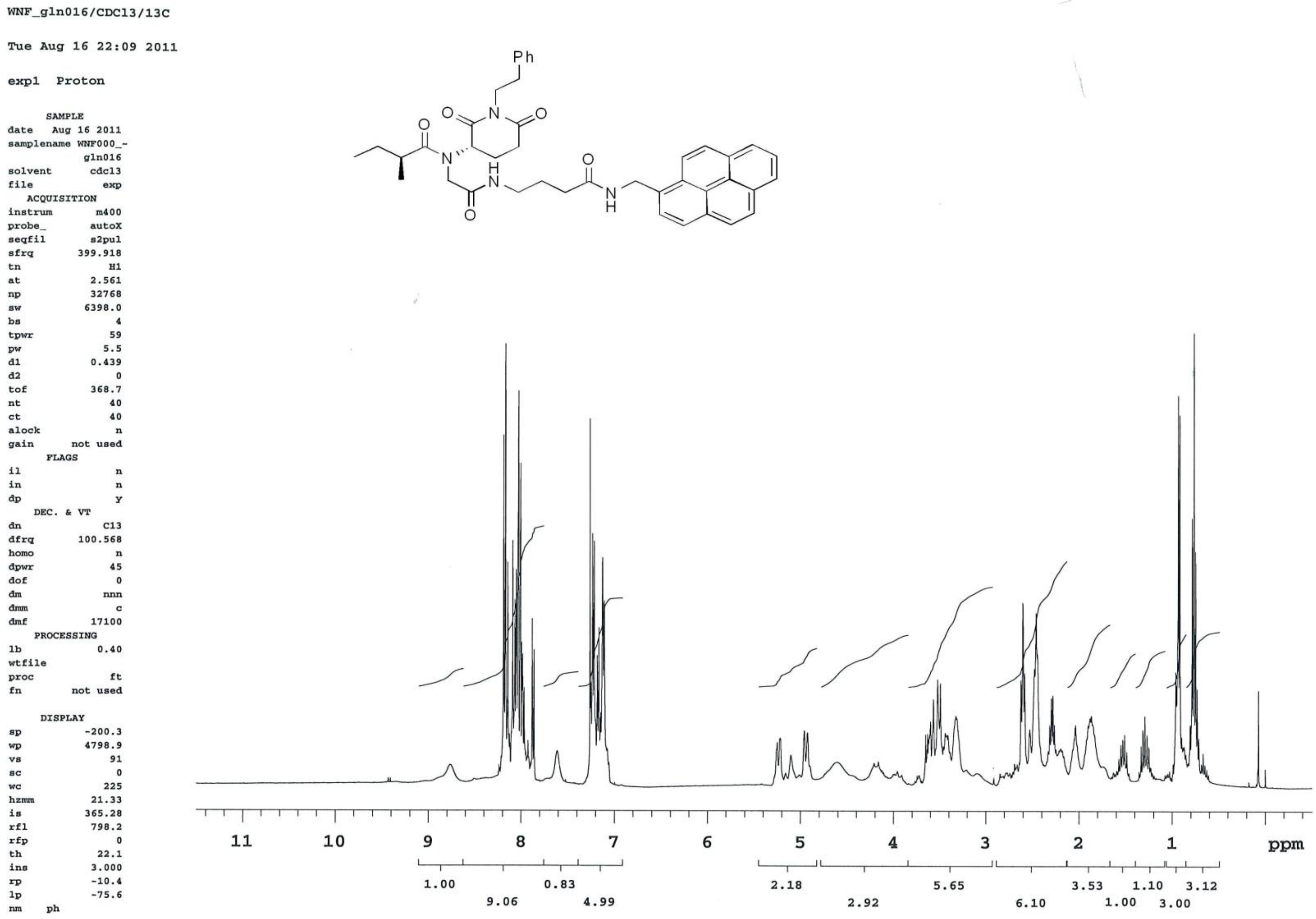

Figure S27: ^{1}H NMR spectrum of compound **10**.

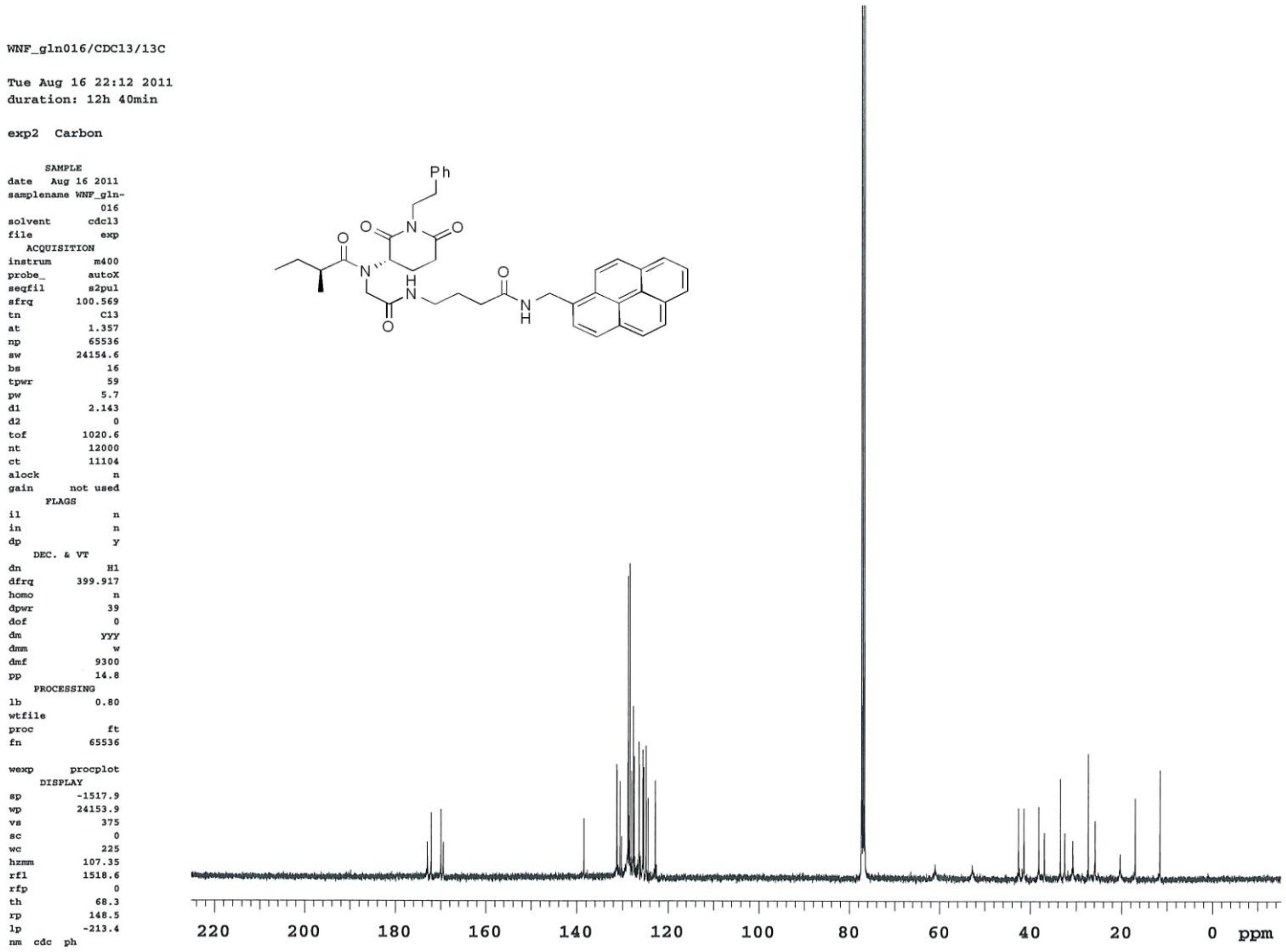

Figure S28: ^{13}C NMR spectrum of compound **10**.
